# Emerging Approaches to DNA Data Storage: Challenges and Prospects

**DOI:** 10.1021/acsnano.2c06748

**Published:** 2022-10-18

**Authors:** Andrea Doricchi, Casey M. Platnich, Andreas Gimpel, Friederikee Horn, Max Earle, German Lanzavecchia, Aitziber L. Cortajarena, Luis M. Liz-Marzán, Na Liu, Reinhard Heckel, Robert N. Grass, Roman Krahne, Ulrich F. Keyser, Denis Garoli

**Affiliations:** † Istituto Italiano di Tecnologia, via Morego 30, I-16163 Genova, Italy; ‡ Dipartimento di Chimica e Chimica Industriale, Università di Genova, via Dodecaneso 31, 16146 Genova, Italy; § Cavendish Laboratory, University of Cambridge, JJ Thomson Avenue, Cambridge CB3 0HE, U.K.; ∥ Institute for Chemical and Bioengineering, ETH Zurich, Vladimir-Prelog-Weg 1, 8093 Zurich, Switzerland; ⊥ Technical University of Munich, Department of Electrical and Computer Engineering Munchen, Bayern, DE 80333, Germany; # Dipartimento di Fisica, Università di Genova, via Dodecaneso 33, 16146 Genova, Italy; ■ Center for Cooperative Research in Biomaterials (CICbiomaGUNE), Basque Research and Technology Alliance (BRTA), Paseo de Miramón 194, 20014 Donostia-San Sebastián, Spain; ○ Ikerbasque, Basque Foundation for Science, 48009 Bilbao, Spain; △ Biomedical Research Networking Center in Bioengineering, Biomaterials and Nanomedicine (CIBER-BBN), Av. Monforte de Lemos, 3-5. Pabellón 11. Planta 0, 28029 Madrid, Spain; ▼ Second Physics Institute, University of Stuttgart, 70569 Stuttgart, Germany; ⬡ Max Planck Institute for Solid State Research, 70569 Stuttgart, Germany

**Keywords:** DNA, data storage, sequencing, random access, error correction, DNA nanostructure, DNA preservation, reading, decoding, costs

## Abstract

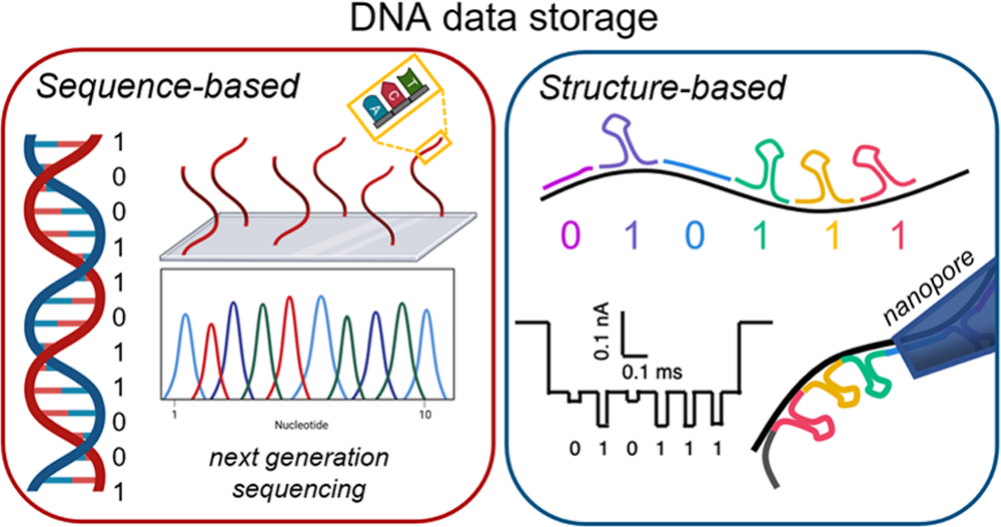

With the total amount of worldwide data skyrocketing, the global data storage demand is predicted to grow to 1.75 × 10^14^ GB by 2025. Traditional storage methods have difficulties keeping pace given that current storage media have a maximum density of 10^3^ GB/mm^3^. As such, data production will far exceed the capacity of currently available storage methods. The costs of maintaining and transferring data, as well as the limited lifespans and significant data losses associated with current technologies also demand advanced solutions for information storage. Nature offers a powerful alternative through the storage of information that defines living organisms in unique orders of four bases (A, T, C, G) located in molecules called deoxyribonucleic acid (DNA). DNA molecules as information carriers have many advantages over traditional storage media. Their high storage density, potentially low maintenance cost, ease of synthesis, and chemical modification make them an ideal alternative for information storage. To this end, rapid progress has been made over the past decade by exploiting user-defined DNA materials to encode information. In this review, we discuss the most recent advances of DNA-based data storage with a major focus on the challenges that remain in this promising field, including the current intrinsic low speed in data writing and reading and the high cost per byte stored. Alternatively, data storage relying on DNA nanostructures (as opposed to DNA sequence) as well as on other combinations of nanomaterials and biomolecules are proposed with promising technological and economic advantages. In summarizing the advances that have been made and underlining the challenges that remain, we provide a roadmap for the ongoing research in this rapidly growing field, which will enable the development of technological solutions to the global demand for superior storage methodologies.

## 1 Introduction

In the present digital era, the quantity of data being produced continues to increase exponentially, with the global demand for data storage expected to grow up to 1.75 × 10^14^ GB by 2025 and by a further order of magnitude within the end of this decade.^1^ The demand for denser and longer-lived information storage devices is also increasing.^2^ Current storage technologies, including optical and magnetic devices, are reaching their information density limits and are thus not suitable for long-term (>50 years) storage, which means that valuable information needs to regularly be transferred to newer storage media if it is to be preserved for future generations. Innovative methods are required for long-term information storage to circumvent this laborious and costly process and to combat other pitfalls associated with current storage media (including energy consumption and insufficient data density).^3^

Nature provides an inspiring example of how to encode, transmit, and preserve information by using DNA to store all genetic information in the form of a four nucleotide sequence. As evidenced by DNA’s invaluable role in the perpetuation of genetic information, these molecules are stable for thousands of years under suitable storage conditions;^4^ for example, 300 000-year-old mitochondrial DNA from a bear has been successfully sequenced.^5^ This DNA sample was preserved in bone, thereby demonstrating that the required power consumption for the archival storage of DNA is very low—another benefit compared with traditional data storage media. In addition to its stability and low cost of storage, DNA presents a major key advantage compared with existing data storage devices: data density. On the basis of its physical dimensions, DNA has a theoretical data density of 6 bits for every 1 nm of polymer, or ∼4.5 × 10^7^ GB/g,^6^ which is orders of magnitude higher than the densities achievable using traditional devices.^7,8^

**Figure 1:**
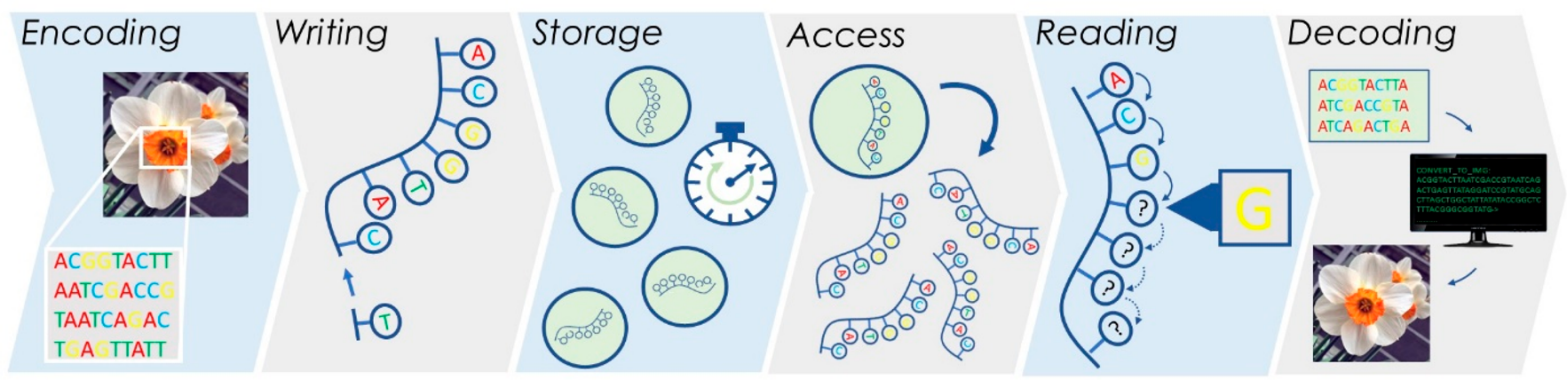
General strategy for DNA data storage, wherein the data is stored directly in the sequence of the oligonucleotides. The six main steps—encoding, writing, storage, access, reading, and decoding—are depicted.

Significant advances have been made in recent years toward using DNA as a digital information storage medium.^9−14^ Existing strategies to encode arbitrary information into DNA do so by translating the desired data (i.e., a movie, book, or picture) directly into the nucleotide sequence, which means that to write each data string, chemical DNA synthesis is employed.^15^ In sequence-based DNA data storage, the major steps comprise: (1) encoding digital information, (2) data writing (synthesis of new oligonucleotides), (3) storing the DNA in physical or biological conditions, (4) random access, (5) data readout via DNA sequencing, and (6) decoding the DNA sequences back into the original digital code, as represented in Figure 1.^8,12^ Over the past decades, substantial advances in biotechnology have significantly bolstered DNA data storage technologies. These include chemical and enzymatic DNA syntheses,^16,17^ polymerase chain reaction (PCR) for DNA amplification,^18^ and DNA sequencing.^19^ Although none of these technologies was initially designed with digital data storage in mind, these considerable developments now enable procedures for writing, random accessing, reading, and editing of data encoded in DNA sequences.^10,11^

**Figure 2:**
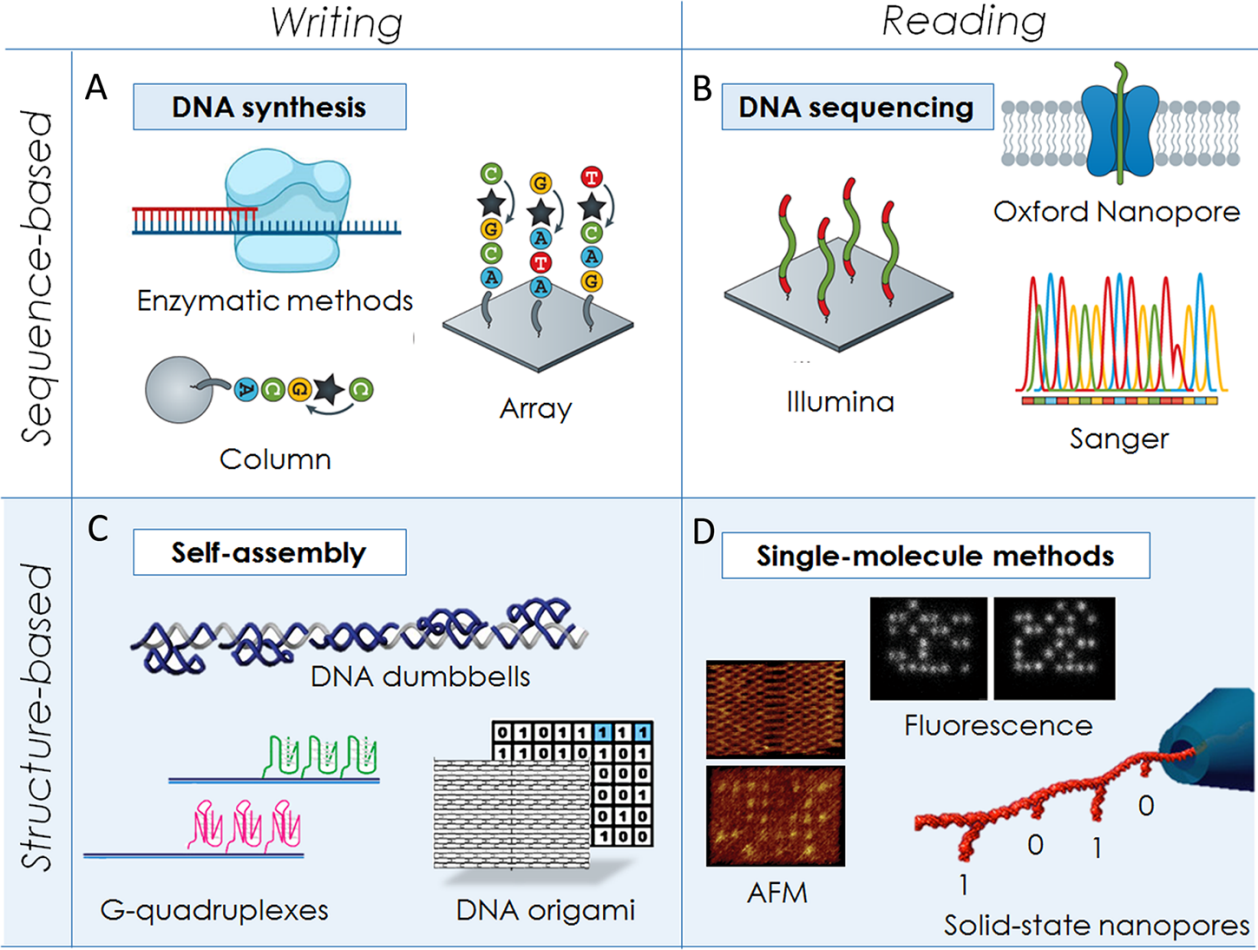
Comparison of the main differences between sequence-based (A,B) and structure-based DNA data storage (C,D), as has been presented in the literature to date. (A,B) Sequence-based storage relies on the *de novo* synthesis of DNA strands and the subsequent sequencing of these entities is performed using next-generation methods. Image adapted with permission from ref (12). Copyright 2019 Springer Nature. (C) By contrast, structure-based methods utilize self-assembly, which means that the information is encoded into their three-dimensional shape. Images adapted with permission: ref (21), copyright 2016 Springer Nature; ref (22), under a Creative Commons Attribution 4.0 License (CC BY), copyright 2021 Springer Nature. (D) These shapes can then be read off using single-molecule methods, including fluorescence, atomic force microscopy, and nanopore techniques. Image adapted from ref (23). Copyright 2019 American Chemical Society.

However, each of the procedures involved in DNA data storage—encoding, writing, storage, random access, reading, and decoding—has significant technical limitations that render DNA data storage, at present, not competitive with magnetic and solid-state storage devices. Because the *de novo* synthesis of long sequences of DNA remains challenging,^20^ these sequences must be broken into smaller fragments (∼200 bases), which requires massive numbers of unique DNA sequences to be made. Data readout also presents several challenges: while in theory analogous to the magnetic readout of a hard disk drive, DNA sequencing must be employed to read out the information stored in individual oligonucleotides. Sequencing often relies on fluorescence outputs, which require expensive fluorophores, optical equipment, and trained personnel, as well as substantial amounts of DNA and long reading times (Figure 2a,b). Nanopore methods may present an appealing alternative, as detailed further in this review. With the use of current technologies, DNA storage is estimated to cost 800 million USD per one terabyte of data (by contrast, tape storage costs approximately 15 USD per terabyte).^12^ The high price of writing DNA data using existing methods prohibits its mainstream adoption as an information storage material.

One potential strategy to circumvent these pitfalls is to rely on the programmable three-dimensional structure of DNA as opposed to its primary sequence (Figure 2c,d). DNA nanotechnology harnesses the specific base-pairing properties of the nitrogenous bases to create arbitrary two- and three-dimensional shapes.^24^ It is possible to generate well-defined, custom objects at the nanoscale using these methods. Information can thus be stored in the 3D structures of these assemblies instead of in the sequence, with readout relying on imaging techniques, such as super resolution imaging,^22^ or using single-molecule nanopore measurements.^23,25^ The structure-based strategy may reduce the number of DNA sequences that must be synthesized by allowing for the erasing and rewriting of data through simple self-assembly. These structure-based methods also eliminate the need for next-generation sequencing, which remains among the most time-consuming aspects of DNA data storage. Because DNA nanotechnology-based approaches capitalize on the self-assembly of DNA sequences, the resulting structures are inherently reconfigurable, which enables data erasing and rewriting without further synthesis.^26^ Moreover, the dynamic nature of these assemblies can be exploited to perform data operations,^13,27^ which allows DNA data storage to integrate directly into the field of DNA computation.

In this review, we provide a detailed description of the two aforementioned methods, which we will refer to as “sequence-based” and “structure-based” DNA data storage. A comparison between them that highlights both the similarities and differences in these approaches will provide an overview of the state of the art in DNA data storage. Finally, we also highlight the exciting potential applications of DNA data storage and manipulation, including archival storage, barcoding, cryptography,^11^ and DNA computing. Despite the hurdles that must be surmounted to implement DNA data storage, it is important to remember that DNA plays an irreplaceable role in biological systems. As such, DNA will never become obsolete as a data storage medium. We posit that the fundamental nature of DNA, in combination with the high density and low energy cost of DNA data storage, will continue to fuel research in this rapidly growing domain.

## 2 Sequence-Based DNA Data Storage Methods

### 2.1 From Encoding to Data Writing in DNA Data Storage

Any digital data (files of any kind such as text and pictures) can be represented as a sequence of bits (i.e., zeros and ones). One possible data storage approach is to use a set of DNA sequences of 60–200 nt in length. The limitations in sequence length arise from the chemical synthesis of DNA; producing DNA strands longer than a few hundred nucleotides (nt) introduces a significant number of errors into the sequence.

**Figure 3:**
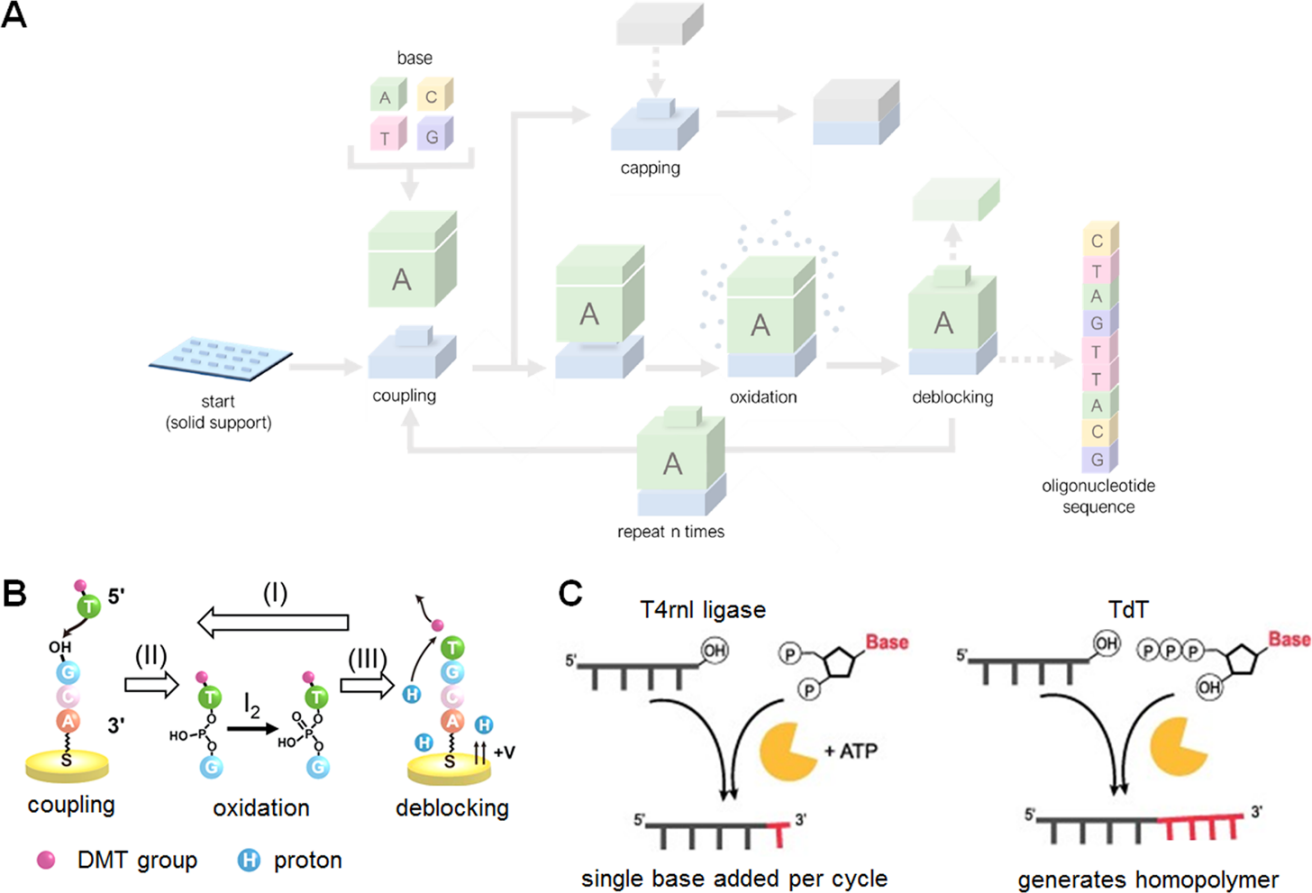
An overview of chemical and enzymatic strategies to synthesize custom DNA sequences. (A) Phosphoramidite synthesis—the most widely used chemical strategy for the synthesis of DNA—involves the sequential addition of nucleotides to a growing chain anchored on a solid support. Protecting groups are employed to ensure that no more than one nucleotide is added at each step and are then subsequently removed via chemical deblocking. (B) Deblocking can also be performed by electrochemistry. Reproduced with permission under a Creative Commons Attribution 4.0 License (CC BY-NC) from ref (31). Copyright 2021 AAAS. (C) Enzymatic methods relying on T4rnl ligase or TdT can also be used to specifically add bases to a growing oligonucleotide in aqueous environments, which eliminates the need for organic solvents. Image reproduced with permission under a Creative Commons Attribution 4.0 License (CC BY) from ref (32). Copyright 2021 Elsevier B.V.

Once properly encoded, data are written on synthetic DNA sequences (Figure 3). Organic chemistry has presented us with a large set of techniques for synthesizing DNA and, as previously mentioned, strands up to 200 nt in length can be readily synthesized. The synthesis is typically performed using phosphoramidite chemistry, which is a four-step cyclic reaction involving the addition of the desired nucleotide to a growing oligonucleotide chain immobilized on a solid support (Figure 3A,B).^28^ The use of a solid support enables extensive parallel synthesis, as well as automation of the chemical process, which will be fundamental to the adoption of DNA for data storage applications.^29,30^ While there are many advantages to phosphoramidite synthesis, it is worth noting that it requires the use of anhydrous solvents, which produce toxic waste.

An alternative to chemical synthesis is enzyme-based methods, but they are still in their infancy. So far, only tiny amounts of data (hundreds of bits) have been stored using enzymatic synthesis versus data consisting of billions of bits using phosphoramidite synthesis. The concept of enzymatic DNA synthesis arose from the discovery of specific DNA polymerases, and this approach is expected to become both cheaper and faster than phosphoramidite synthesis for data storage applications.^40^ A major limitation, however, is DNA polymerase’s need for a template strand. To create a user-defined DNA sequence as in the chemical method, enzymes capable of extending the 3′ end of the ssDNA in a template-independent manner, such as polynucleotide phosphorylase (PNPase), T4 RNA ligase, and terminal deoxynucleotidyl transferase (TdT, Figure 3D), are required.^32^ In particular, the use of TdT, a template-independent polymerase, to synthesize DNA oligonucleotides was shown to be a promising alternative to chemical synthesis.^33,16^ Among others, Lee et al.^16^ reported on a technique for enzymatic synthesis and digital coding that was based on template-independent polymerase TdT and nanopore reading. This strategy allowed the archiving of information in DNA without mandatory single-base precision, as well as cost reduction due to miniaturization and enzyme recycling. Moreover, the synthesis of 1000-nucleotide-long strands with homopolymeric stretches enabled a reduction of the synthesis time (Figure 3D). Palluk et al.^28,33^ also described an oligonucleotide synthesis strategy that uses TdT and demonstrated that TdT–dNTP conjugates can quantitatively extend a primer by a single nt in 10–20 s. Crucially, this scheme can be iterated to write a user-defined sequence. Compared with chemical synthesis, which is undertaken in organic solvents, the enzymatic synthesis is compatible with aqueous conditions.

Both chemical and enzymatic syntheses are severely limited by the low speed of these processes.^29,39^ Achievement of the necessary parallel writing capabilities while maintaining a realistic infrastructure footprint requires maximization of the number of different sequences that can be synthesized per unit area, simultaneously, on a single platform. The most space-efficient way to increase synthesis density is to reduce the area over which each unique sequence is grown (the feature size), the distance between features (the pitch), or both. To this end, photomask arrays have proven to generate high oligonucleotide densities;^34^ however, this technique relies on a series of bespoke photolithographic masks to synthesize a defined set of sequences, that is, masks must be created for each set of desired sequences. An alternative method uses electrode arrays and leverages the scaling and production roadmap of the semiconductor industry, where features as small as 5 nm are now common. For example, Nguyen et al.^35^ produced an electrode array and demonstrated independent electrode-specific control of DNA synthesis with electrode sizes and pitches that enabled a synthesis density of 25 million oligonucleotides/cm^2^ (Figure 3C). Finally, the printing synthesis method has rapidly become the most applied method (also thanks to commercial technological platforms, such as Agilent and Twist).

The sequences to be synthesized are defined by the encoding process, which maps the data to a set of DNA sequences so that a corresponding decoder can reconstruct the information, even though the writing, reading, and storage of the DNA introduces errors.^9,14,36,7,37,29,38,16,39−41^

DNA storage systems overcome these errors without losing data by capitalizing on both physical and logical redundancy. Physical redundancy is achieved by creating many, sometimes inaccurate, copies of each sequence, which enables a consensus to be reached when the data is read. Some errors cannot be resolved using physical redundancy alone. Logical redundancy guarantees reconstruction even when errors occur. While physical redundancy occurs automatically during the synthesis process—many copies of each sequence are always produced—it is fundamental to apply dedicated algorithms to include logical redundancies in the initial encoding. Moreover, encoding and decoding are strictly connected. The algorithms that encode the data to be stored add redundancy in a principled way so that a decoding algorithm can reconstruct the data from noisy reads (Figure 3A).

During the 2010s, extensive innovations in algorithm development have enabled reliable storage of data even under significant errors. Grass et al.^4^ used modern error-correcting codes in the context of DNA storage, and a variety of different schemes have been proposed.^4,42−50,36,14^ While physical and logical redundancy lower the storage density of DNA, recent works have proposed to raise it by expanding the DNA alphabet using composite natural letters^7,51,52^ or chemically modified nucleotides.^53^

### 2.2 Storage and Degradation Issues

Despite DNA’s long-term stability in well-controlled environments such as ancient bone, with storage durations as long as several hundred thousand years,^54,55^ both aqueous solutions and dried DNA only exhibit a half-life on the order of months to a few years under ambient conditions.^56^ Therefore, considerations for the physical storage of data-encoding DNA are crucial for realizing its potential for long-term data storage. Without appropriate protection, DNA (and thus the data encoded within) is degraded by multiple mechanisms, including strand breaks, nucleotide mutations, strand cross-linking by UV, oxidation, hydrolysis, alkylation, or mechanical stress, all of which are due to environmental factors. Among those, hydrolysis is the dominating decay pathway in a data storage context.^57,58^ Thus, all applicable DNA storage approaches focus on protecting the DNA from moisture and oxygen with either microscopic (i.e., on the level of individual molecules) or macroscopic (i.e., on the level of individual pools) containers. Examples of microscopic containers include encapsulation within silica particles;^56,59−61^ embedding in alkaline salt,^62^ polymer,^63^ sugar,^64^ or silk protein^65^ matrices; and coprecipitation with calcium phosphates^66^ imitating bone. In the latter category, dried or lyophilized DNA is stored on filter paper^64^ within hermetically sealed capsules with inert atmosphere^57,58,67,68^ or, as is common in biological practice, simply frozen in aqueous solutions and stored at −20 or −80 °C.^69^

Generally, all storage approaches trade long-term stability with a decrease in storage density by 1–3 orders of magnitude, caused by the low loading ratio between DNA and carrier (see Table 1). Additionally, the required time and cost for protection can be a distinguishing factor for DNA data storage systems, albeit less so for long-term storage applications.^69^ The size of a single DNA pool is an important consideration for the design of DNA storage media, as index sizes for random access, constraints of PCR, and required physical redundancy for retrieval imply an upper limit on the number of pooled oligos.^69,6^ This represents the maximum data that can be stored within a single macroscopic storage container, and has been estimated to lie between a few TB up to a few hundred TB.^56,6,70^ We compared the storage densities and half-lives of micro- and macroscopic storage approaches in Table 1 by using the largest model pool size for which random access has been demonstrated at 5.5 TB.^71^

**Table 1:** Comparison of the DNA loadings (g DNA/g carrier), achieved information density in PB/g, and extrapolated half lives for both macroscopic and microscopic storage approaches, with an assumed pool size of 5.5 TB.^71^_;_^a^

storage approach	τ(10 °C)/ years	DNA loading^c^	density^c^/PB/g	references
macroscopic				
in solution^b^	17	0.005%	0.85	(57)
dried	7	100%	17 000	(66)
bone	1700	0.05%	8.5	(54,62)
DNAshell^c^	>100 000	0.000 02%	0.0034	(58,68)
microscopic				
trehalose matrix	160	0.13%	2.2	(64)
silica particles	540	3.4%	580	(59,4)
polymer matrix^d^	110	0.1%	17	(63)
salt matrices	750	20%	3400	(62)
silk matrix^e^	NA	0.000 03%	0.0051	(65)
calcium phosphate matrix	600	18%	3060	(66)

a All values are considered at 10 °C and assuming DNA with 150 bp at an information density of 17 EB/g. Temperature corrections were performed using Arrhenius Law using 155 kJ/mol as the activation energy of DNA strand breaks.^57,4^

b Typical concentration for synthetic DNA is 500 ng/μL.

c Assumed pool size per DNA shell = 5.5 TB.^71^ Weight of the DNA shell is at least 1.3 g.^58^

d Polymer density was assumed as similar to that of polyethylene glycol at 1.12 g/cm^3^.

e Density of filter paper is around 85 kg/m^2^.

Current approaches towards data encoding in DNA, such as the use of altered DNA topology^23,72^ and third-generation sequencing platforms, present new challenges to data storage, as those approaches rely on oligos with multiple hundreds to thousands of nucleotides in length, compared with the few hundreds of nucleotides commonly in use for next-generation sequencing (NGS).^70^ While both micro- and macroscopic storage systems are independent of sequence length, DNA decay by hydrolysis scales with the number of nucleotides per oligo and, thus, a proportional increase in the expected number of errors is anticipated.^73^ Given that some types of single-site errors, such as strand breaks, may render entire oligos and the data within unreadable, the use of longer sequences further increases the need for durable storage to prevent premature data decay beyond experimental time scales. To this end, systematic studies on decay mechanisms and rates for many approaches to data encoding in DNA are missing, a critical factor regarding approaches that heavily rely on structural integrity for data retrieval.

Currently, long-term storage is only feasible within a protective material and at DNA loadings of only a few percent. Consequently, the need remains for long-term DNA data storage systems closer to DNA’s true storage density. Indeed, further improvements in the coding density toward DNA’s Shannon capacity, for example, by means of improved encoding algorithms or lowering logical redundancy, are largely overshadowed by the general loss of storage density due to the storage matrix. Conversely, the loss in encoding density yielded by encoding approaches relying on DNA topology is rendered less severe by this storage overhead, and the interplay of such approaches with denser storage systems is interesting for further research.

### 2.3 Random Access

**Figure 4:**
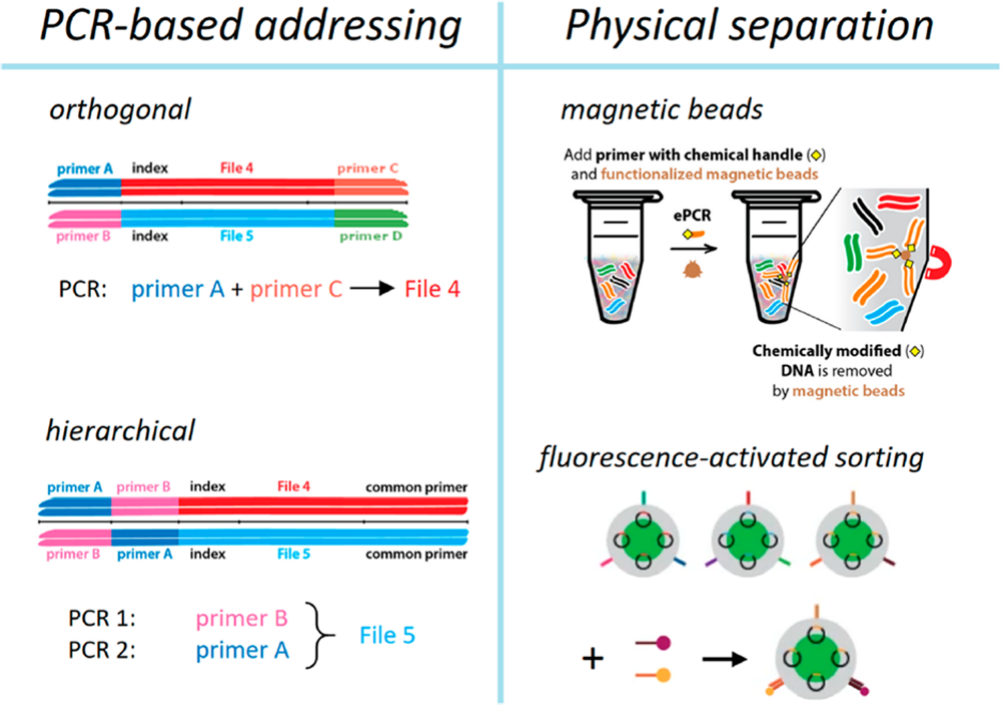
Overview of random access strategies to select a subpool of sequences, usually a file, from a large pool. PCR-based addressing methods leverage the high specificity of primers and the exponential amplification of PCR to enrich target sequences by using either a single or multiple PCR runs. Methods using physical separation as a tool to select sequences also rely on the high specificity of short primers or barcode sequences, but remove the desired sequences using magnetic bead extraction or fluorescence-activated sorting. Images adapted from ref (71) and reproduced with permission from ref (75). Copyright 2019 American Chemical Society and copyright 2021 Springer Nature, respectively.

As discussed above, the ability to select only a subset of DNA molecules for readout limits the current data capacity of a single pool of data-encoding DNA. This access to DNA subpools, equivalent to file-level random access, is crucial to scale DNA-based data storage up to large data capacities with no need for costly, complete sequencing of the pool. This has a major implication: an addressing system is needed to select subpools from a complex DNA mixture, with high specificity. Whereas the use of a physical substrate on which DNA can be arrayed may solve this problem,^31^ this approach and similar solutions relying on the physical separation of individual oligos or oligo pools render DNA’s density advantage obsolete. Instead, two other major strategies have been developed: PCR-based addressing and direct physical separation (Figure 4). In PCR-based addressing systems, the high specificity of amplification via PCR is leveraged to selectively enrich a subpool over the background by using at least one address-specific primer and corresponding priming regions on the data-encoding oligos. Because of PCR’s exponential nature, a sample of the amplified pool will contain mainly the desired file with its matching priming regions, as well as nonspecific sequences as background. Demonstrated in 2015,^74^ this addressing system has now been shown to scale to well above 10^10^ unique sequences per reaction while only requiring about 10 copies per sequence, which is equivalent to a pool capacity on the order of terabytes.^6,70,71^ Either a rigorous design of orthogonal primer sequences^6^ or the use of hierarchical addressing systems would be needed to achieve the required high specificity at these scales.^71^ Nonetheless, primer-based addressing systems face several constraints. First, the incorporation of random-access priming regions into each oligo decreases the available space for data-encoding bases, thereby also decreasing the storage density (currently by about 15% per address region).^71,75^ Second, PCR-based random access irreversibly removes oligos from the pool, which necessitates potentially lossy reamplification of the entire pool after repeated data retrieval.^75,76^ Moreover, as pool sizes and, thus, the number of sequences, become larger, the enrichment of a few copies against an ever-increasing background will at some point hit the limitations of PCR regarding processing volumes, required amplification cycles to obtain sufficient enrichment, and nonspecific amplification due to primer–payload similarity.^77,70^ Indeed, data retrieval from a hierarchical addressing system of 5.5 TB required additional physical separation of pools via a biotin-based bead extraction between file accesses to fully remove the background carried over from PCR.^71^ Lastly, PCR-based addressing is incompatible with common storage approaches, thus necessitating the removal and re-embedding of the encoding DNA into the storage matrix for each random-access operation.

As an alternative to PCR, sequence specificity has also been exploited to carry out physical separation of files in pools. As mentioned above, biotin-labeled primers can be used to address and extract specific files via streptavidin magnetic beads on the basis of file-specific random access regions in encoding oligos, similarly to PCR-based addressing.^71,78^ This approach has two key advantages: the sample can be reused for subsequent retrievals and nonspecific binding and PCR-induced biases are circumvented.^78^ Banal et al.^75^ extended this concept to DNA pools encapsulated in silica particles by labeling their surface with DNA barcodes to facilitate random access via fluorescently labeled probes and fluorescence sorting. While this represents a scalable random access scheme compatible with long-term storage, it is likely that the DNA barcodes on silica particles would decay much faster than the data-encoding DNA within so that random access ceases to function even if the data itself may still be intact.

All random access approaches aim at facilitating file-level control in large pools of DNA-encoded files while under the constraints of specificity, scalability, and storage density. Such scaling to large pools is highly desirable because it retains DNA’s high storage density compared with the physical separation of smaller pools using storage approaches (see Section 2.2). Currently, the highest demonstrated data capacity for random access is on the order of terabytes of data.^6,12,71^ While this does not appear to be a hard limit,^6^ it is likely unpractical to scale random access by PCR-based addressing indefinitely because of the aforementioned difficulty of orthogonal primer design and the requirement for many amplification cycles given the associated impact of PCR bias.^20^ Whether any practical limit of PCR-based random access exists in real-life applications remains to be seen, however. As an alternative, hierarchical storage systems combining high-level access to isolated subpools with file-level random access within such subpools appear more suited to allow for random access at the data capacities envisioned for DNA data storage. The first steps in this direction have been taken, such as labeling DNA-embedding polymer disks with QR codes or automated retrieval of individual DNA pools in a digital microfluidic device,^56,63^ but the trade-off between storage density, data longevity, and ease of automated data access requires further work.

Beyond random access, other file operations such as encryption with genomic keys,^79^ erasure on the basis of obfuscation,^80^ and rewriting by chemical modification or PCR^81,82^ are also supported by sequence-based DNA data storage. As recently reviewed elsewhere,^83^ these approaches highlight the versatility of file operations supported by DNA as a storage medium.

### 2.4 Reading

**Figure 5:**
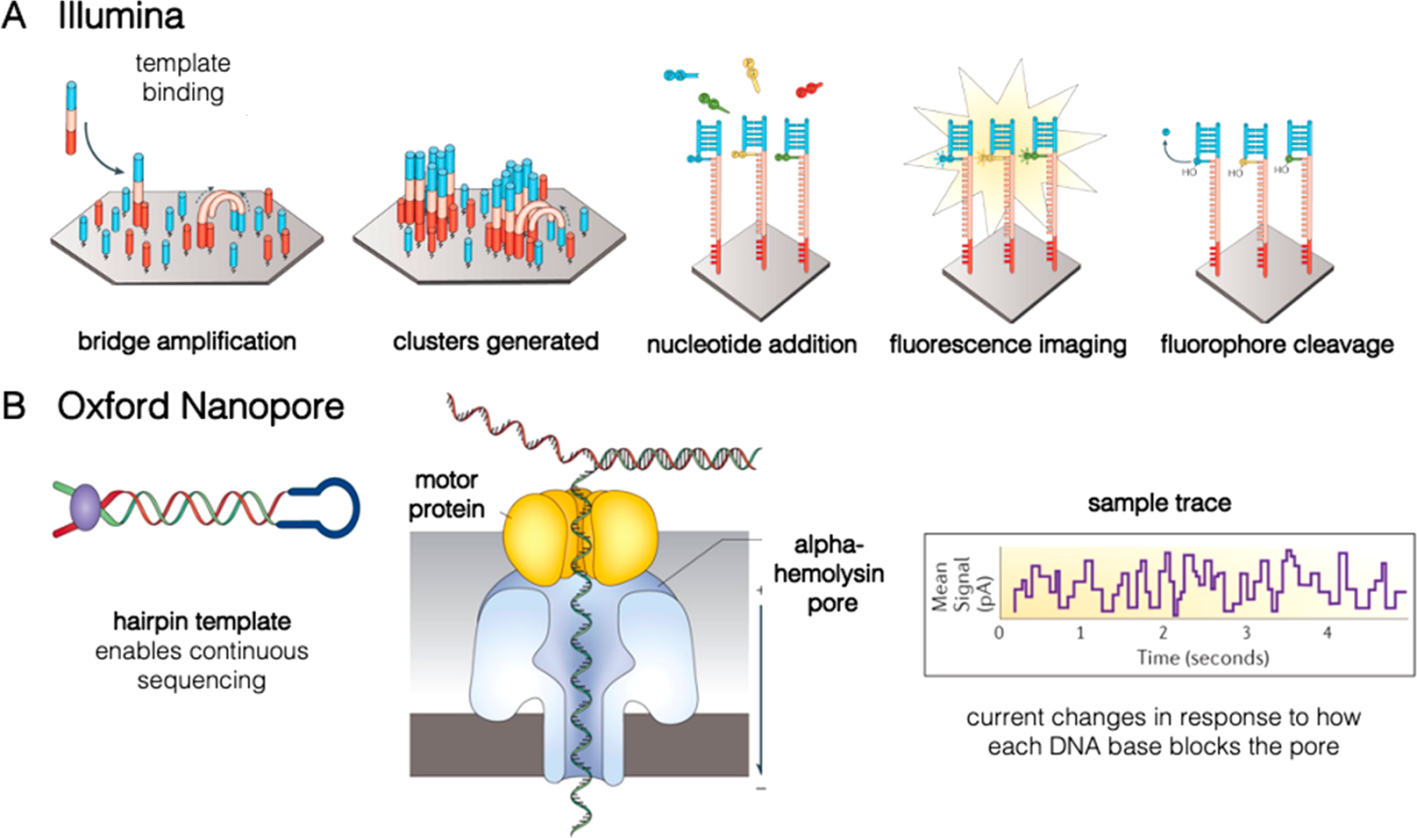
Overview of next-generation sequencing technologies presently used in DNA data storage. (A) Illumina sequencing generates clusters of identical single-stranded oligonucleotides. As the complement is synthesized using spectrally distinct, fluorescently tagged nucleotides, the identity of each base along the strand can be determined through the color of emission. (B) Oxford Nanopore measurements do not require fluorescent dye molecules. As the oligonucleotide passes through the protein pore, the three-dimensional shape of each base will modulate the ionic current, which results in a current–time trace that corresponds to the specific sequence. Images adapted with permission from ref (85). Copyright 2016 Springer Nature.

While the readout of data encoded in DNA is rarely done in its application as an archival storage system,^69^ the complete and error-free retrieval of stored data must be guaranteed within a defined set of storage and sequencing conditions in order for DNA data storage to have any commercial relevance. As a result, the choice of sequencing platform has a marked impact on the design and feasibility of sequence-based DNA data storage. Currently, readout of the DNA sequences needed for data decoding relies heavily on established technologies for DNA sequencing in life science applications, most prominently sequencing-by-synthesis (SBS) as commercialized by Illumina.(Figure 5A,B).^84,85^ As an alternative, sequencing using protein nanopores, commercialized by Oxford Nanopore Technologies, has been used because of its ease of implementation, automation, and portability (Figure 5C).^70,81,86^ Nanopore sequencing uses electrical readouts rather than fluorescence detection to identify each base of a DNA strand as it moves through a biological nanopore. Contrary to SBS, it is therefore also able to identify modified and unnatural nucleotides such that the readout of data encoded using an expanded molecular alphabet is possible.^53,87^

While nanopore sequencing improves upon several limitations of SBS for DNA data storage, as reviewed by Ceze et al.,^12^ two key constraints of the technology are its high error rate and the required sequence length. The high error rate of nanopore sequencing (∼10% per nt in the single read),^70,88^ compared with the nearly negligible rate of errors introduced by SBS (∼0.5% per nt),^70^ necessitates the clustering of sequence information, and thus, higher sequencing coverage and additional postprocessing of sequencing data.^70,86^ Moreover, sufficient pore utilization for high sequencing throughput can only be realized for long fragments (>1 kb).^86,88^ Therefore, the readily available oligo libraries with a length of only a few hundred nucleotides per sequence must be combined into longer assemblies to be suitable for nanopore sequencing. This process, usually performed via Gibson assembly or overlap extension PCR,^70,81,86^ reintroduces several difficult-to-automatize steps into the sequencing workflow, which calls the approach’s claims of improved portability and ease of automation over SBS into question. These constraints currently render nanopore sequencing more challenging and slower than SBS.^12^ Accordingly, the largest data size retrieved using the technology is currently about 1.67 MB, compared with around 200 MB for SBS.^70,86^

The use of both state-of-the-art SBS and rapidly developing nanopore sequencing for DNA data storage highlights the current trade-off between sequencing accuracy and cost, as well as implications for future scalability. To this end, the development of solid-state nanopores for the determination of DNA structures including their sequence, with the potential of increased accuracy and throughput by avoiding enzymes limiting the translocation rate, holds promise for data storage applications.

### 2.5 Decoding and Error Correction

In addition to the errors during DNA sequencing discussed in the previous section, errors are also introduced during the synthesis, storage, and amplification steps of DNA data storage, which presents challenges regarding data decoding. While amplification and SBS-based platforms mainly introduce substitution errors (reading a C instead of a G, for example), synthesis dominantly causes deletions (e.g., missing a base) at a final rate of around 0.2–1% per nt.^20,62,70^ Insertions (addition of extra bases) are uncommon and usually occur at less than 0.1% per nt, mainly because of synthesis.^20,62^ In addition to biases in amplification efficiency, storage mainly contributes to shifts in the copy number distribution of the sequences, which leads to the unrecoverable loss of individual sequences over time, e.g., 8% after 94% of the DNA has decayed (i.e., four half-lives).^20,25^ This means that, in general, sequence information is never recovered error-free. As the decoding of the stored data directly depends on this sequence information, both the loss of individual sequences and the introduction of errors into these sequences pose a risk on error-free decoding. While an increase of physical redundancy to cluster sequence information alleviates this problem, doing so is undesirable and inefficient because it drastically lowers the information density.^20^ As considerations for cost and automation limit most of the potential for reducing error rates within the data storage workflow, sufficient redundancy must instead be implemented at the sequence level. Therefore, the presence of errors in DNA storage necessitates the use of principled coding/decoding algorithms. The goal of a good encoder/decoder pair is to enable perfect reconstruction from noisy data by introducing a minimal amount of logical redundancy. Error-correcting schemes tailored to DNA data storage consider that the written sequences are relatively short and typically stored in a spatially disordered manner. The optimal coding schemes depend on the noise profile of the storage system. Reliance on logical redundancy introduced by a combination of modern error-correction codes is sufficient for low error rates. However, both for low and large error rates, dominated by deletion errors, one also uses physical redundancy to recover the original information.^42^

An error-correcting code maps an original message to a larger one, which introduces redundancy. If this message is then sent over a noisy channel, thereby introducing random errors, these errors can be detected or corrected. A simple example of an error-correction code was used by Goldman et al.,^9^ where each part of the information was written on four subsequent DNA sequences. Thus, the loss of sequences could be corrected if fewer than four subsequent sequences were lost. This coding scheme, however, was ill-suited for the used DNA channel because it had a low effective information rate, i.e., number of information bits per total number of encoded bits, and did not recover the whole message. In contrast, good error-correcting codes ensure data recovery with minimal redundancy. The maximal information rate that an error-correcting code can achieve is theoretically bounded.^89^ This bound is known as the channel capacity and depends on the characteristics of the noisy channel. This means that the parameters of a good error-correcting code depend on the rates and type of errors. For example, the Reed–Solomon code can correct up to *e* erasures and *s* substitutions with 2*s* + *e* additional symbols.^90^

**Figure 6:**
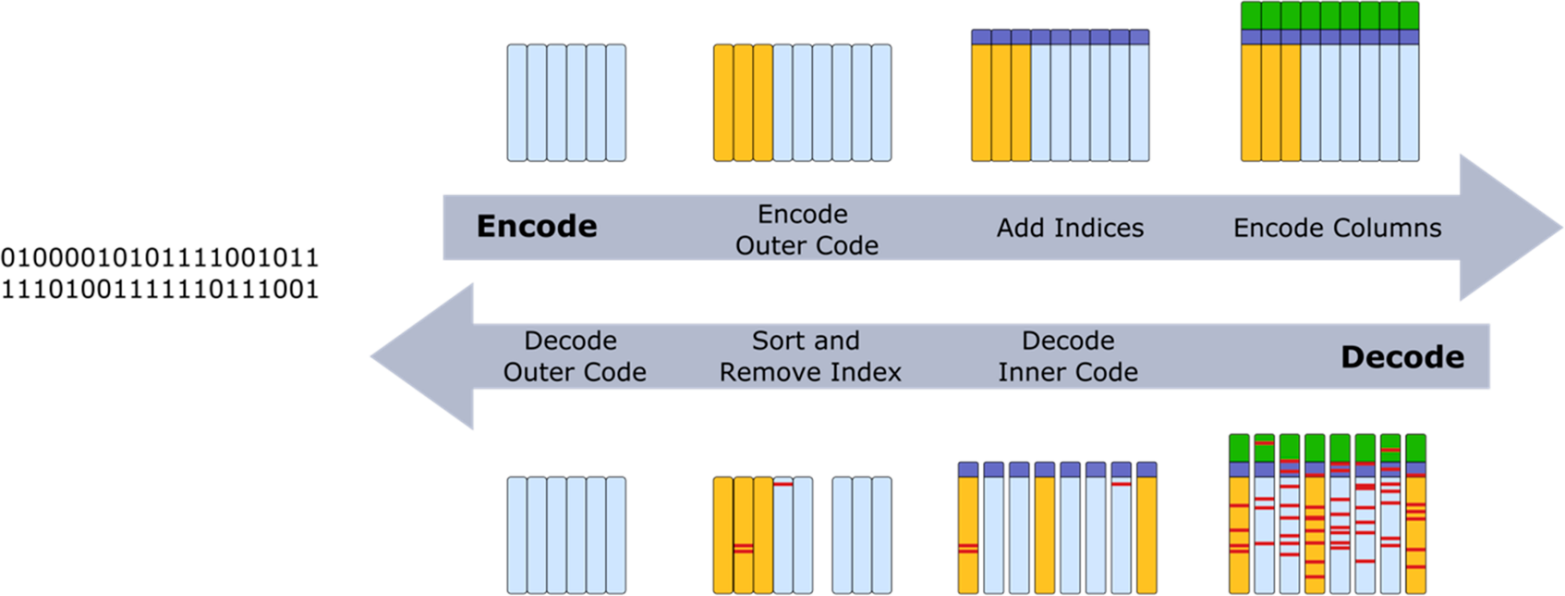
Inner–Outer Code. ***Encoding***. The original information is first encoded with an outer code that introduces redundancy and protects against the loss of sequences. In Grass et al.^4^ the original information was first grouped into blocks of multiple sequences (light blue). Then, each row was encoded with a Reed–Solomon code that adds redundancy (yellow). The columns correspond to single DNA sequences. These are labeled with a unique index (purple). Each column is then encoded with an inner code that adds logical redundancy on the level of each sequence (green). In general, the inner and outer codes need not add the redundancy separate from the original data, but instead return a modified longer word. ***Decoding***. The original information from the set of noisy sequences (errors marked in red) is retrieved by first decoding the inner code. This removes most errors within the sequences. For large error rates dominated by insertions and deletions, this step may be preceded by a clustering and alignment step that generates sequences with fewer errors from multiple noisy copies. The sequences are ordered by their index. The ordered sequences are then decoded by the outer code. Here, lost sequences correspond to erasures and erroneous sequences to substitutions. These are corrected by the outer code.

In 2015, a DNA data storage that used an error-correcting scheme, which enabled the recovery of full data, was realized by Grass et al.^4^ Its encoding/decoding algorithm is explained in Figure 6. It uses an outer code that can correct for the loss of sequences, adds an index for each sequence to be able to retrieve the order of the sequences that are lost during storage, and uses an inner error-correcting code that can correct nucleotide errors within sequences. Following the original introduction of the inner-outer encoding scheme, the vast majority of subsequent works used such a scheme for DNA data storage.^14,42,44,70^ In general, the outer code applies on the level of the original information, whereas the inner code protects single sequences or indices. However, different codes were used for the outer and inner codes. A Reed–Solomon code,^4,44,70^ Fountain codes,^14^ and LDPC (low-density parity check) code were used as an outer code.^91^ As an inner code, a Reed–Solomon code was used by Grass et al. and Organick et al.^4,70^ Blawat et al. proposed to protect the index separately with a bit-correcting code (BCH) as the inner code.^44^ The inner–outer coding scheme works well for moderate error rates of 1–2% and substitutions. However, it cannot correct large error rates that are dominated by insertions and deletions. This is because no inner codes exist that work sufficiently well on short sequences in these noisy setups.^92^ Here, the original message can be recovered by additionally exploiting the physical redundancy. For example, in Antkowiak et al.,^42^ the noisy sequences were first clustered by similarity, then the information on multiple erroneous copies was combined to construct a sequence with fewer errors. This was achieved by an alignment step within the clusters and subsequent majority voting. This resulting sequence could then be sent through the usual decoding steps. Recent works have explored the development of efficient clustering methods tailored to DNA data storage, as well as efficient encoding schemes that allow the recovery of a sequence from multiple noisy reads.^93−96^ Such codes could then be used as an inner code. This has led to a better understanding of efficient use of physical redundancy in DNA data storage. However, at this moment an optimal encoding/decoding scheme for long-term DNA storage, or even components of it, remains unknown. For example, the capacity of deletion and insertion channels or reconstruction from multiple reads with the combination of different errors are not fully understood yet. Also, coding for these short unordered sequences remains challenging for very high error rates. Furthermore, different synthesis and sequencing techniques might motivate different approaches. For these reasons, error correction for DNA remains an active topic of research.

### 2.6 Limitations of DNA Data Storage

#### 2.6.1 Issues Related to Cost

**Figure 7:**
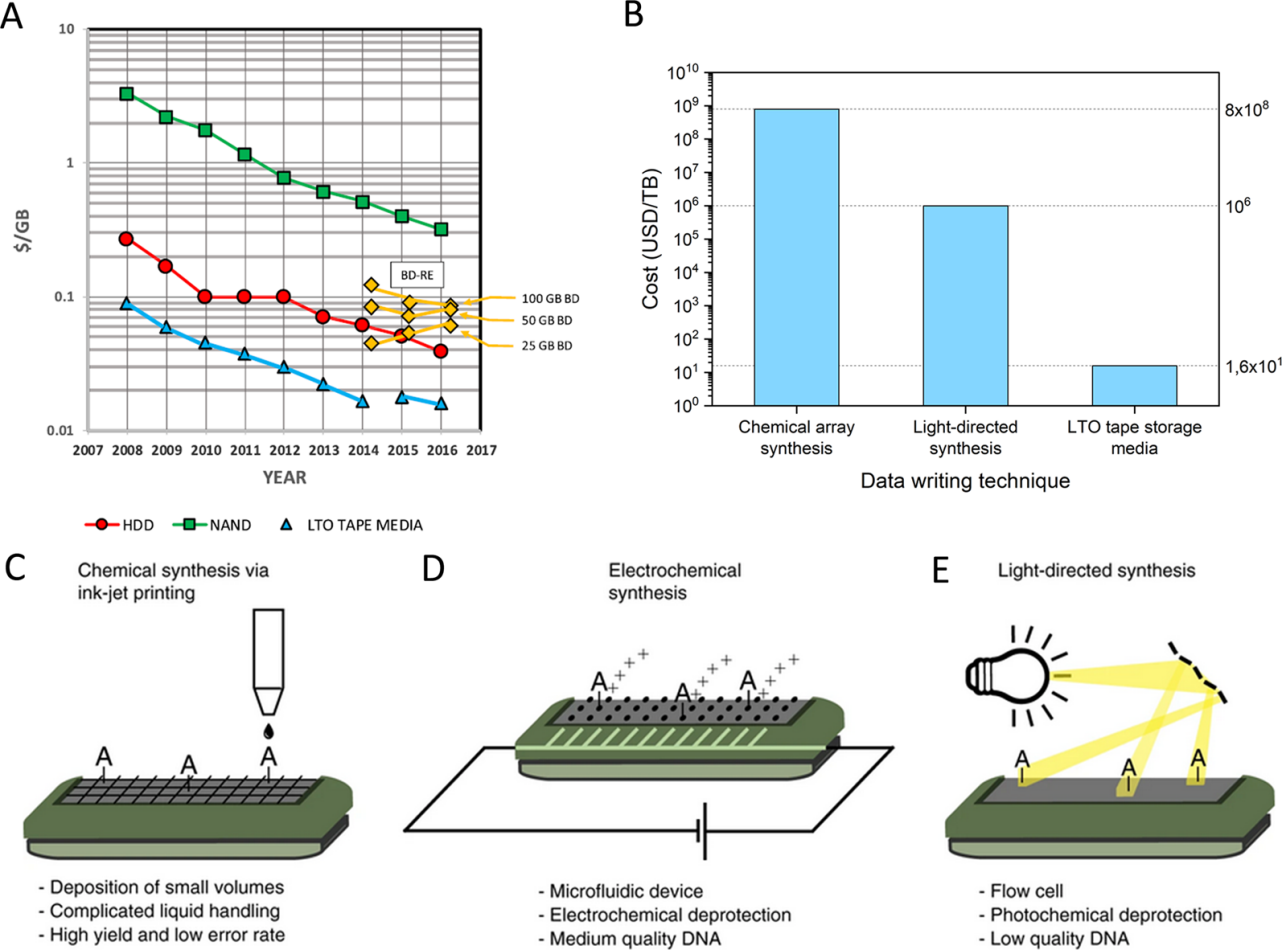
(A) Cost trend of hard disk drives (HDD), NAND flash-based storage devices, linear tape-open tape cartridges (LTO tape), and optical Blu-ray (BD-RE). Image has been reproduced with permission under a Creative Commons Attribution 4.0 License (CC BY) from ref (99). Copyright 2018 AIP Publishing LLC. (B) Cost comparison between DNA synthesis for data storage and LTO tape storage. (C–E) Comparison of different DNA synthesis platforms and their characteristic traits. (C) Printing technology is primarily used by Twist and Agilent. (D) Electrochemical synthesis is employed by Custom Array. (E) Antkowiak et al. used light-directed synthesis. (C–E) Images reproduced with permission under a Creative Commons Attribution 4.0 License (CC BY) from ref (42). Copyright 2020 Springer Nature.

DNA has become a promising tool for next-generation data storage since it provides high data capacity and storage density^78^ and it is possible to store it in multiple ways^37^ over significant time periods.^4^ However, in order to make DNA data storage standard, some limitations must be overcome. Arguably, the most important limit to the development of DNA data storage is cost, especially in comparison with standard storage processes. Often, synthesis costs for DNA data storage are undisclosed;^12^ however, it is possible to draw some conclusions about them. The synthesis of DNA oligos for data storage was column-based and was developed in the 1980s. Since then, this process has been fully automated, and now it allows the synthesis of 96–384 oligos simultaneously. The costs of this procedure range between 0.05 to 0.15 USD per nucleotide.^11^ Array-based synthesis processes were developed in the 1990s. They lowered the costs because of their high-throughput nature, with an average price per nucleotide down to 10^–4^ USD. Thus, if a conservative estimate of 1 bit/nucleotide of encoded data is assumed, each terabyte of digital data would cost 800 million USD, on average.^12,97^ In comparison, tape storage costs 7–8 orders of magnitude less, i.e., about 16 USD/TB of data, with prices decreasing by 10% every year (Figures 7A,B).^12,98^ Considering this enormous disparity in cost between DNA data storage and magnetic tape, the outlook for DNA storage solutions initially appears dismal. That being said, DNA data storage has the potential to drop significantly in cost over time because of several key features. For example, optimized error-correcting codes could lower the cost^97,14^ by increasing the overall efficiency of the storage process by means of accuracy reduction.^12^ By capitalizing on error-correcting codes, it may be possible to work with cheaper, albeit less reliable, synthesis processes if it is assumed that any synthetic errors can be identified and corrected for upon readout, thereby leading to an overall reduction in cost. In 2020, Antkowiak et al. proposed that synthesis costs will drop to around 10^6^ USD/TB (i.e., 2–3 orders of magnitude reduction) as a result of improved synthesis strategies, including large parallelization, optimization of reagents, and combination of nonvolatile DNA-based memories with logical operations (Figures 7B–E).^42^ In addition, Antkowiak et al. estimated the marginal costs of the chemical synthesis of DNA. With the use of photolithography to synthesize 10 000 copies of each oligo, with a nucleotide reagent cost of 100 USD/g and a logical density equal to 1 bit/nucleotide, the cost of 1 TB of data stored in DNA would be ∼10^–2^ USD, with a chemical yield of 100%. Even if this chemical yield is impossible to achieve in industrial conditions, DNA data storage will be competitive against tape storage (20 USD/TB cost) even at 0.1% chemical yield. In the latter case, the cost of photolithographic DNA storage would be ∼10 USD/TB, and synthesis conditions would be similar to the one used in surface chemistry (1000× reagent excess), which demonstrates that an optimization of chemical DNA synthesis processes is compatible with DNA data storage applications. Thus, Antkowiak et al. proved that the combination of synthesis processes that produce lower quality DNA oligos (i.e., photolithographic synthesis) and appropriate error-correction codes allows a major cost decrease in DNA data archives.^42^ Regarding costs, there is also an important advantage with respect to traditional storage technologies that is worth mentioning. In fact, DNA storage systems’ maintenance costs are expected to be lower than the ones of silicon devices in contemporary data centers.^97^

A strategy toward decreasing the costs of stored DNA data may be the enzymatic synthesis of DNA strands.^72^ This synthesis could, in principle, decrease the costs of reagents even if the required enzymes are still rather expensive. It occurs in aqueous environments and it yields longer strands; however, error rates need to be assessed. A brief review of the principal trends in enzymatic synthesis is provided in section 2.1. The costs of enzymatic synthesis have been estimated by Jensen et al. for a template-independent enzymatic oligonucleotides synthesis (TiEOS) method.^100^ The total costs of synthesizing 1000 strands of 1000 nucleotide length would be 136 USD with recycled TdT, 2700 USD by phosphoramidite technique, and 136 000 USD if a fresh stock of TdT was introduced at every cycle. Thus, the costs of the enzymatic synthesis would be 1 order of magnitude lower than the phosphoramidite technique if the TdT was recycled.^100^ The combination of advanced error-correcting codes and synchronization algorithms could possibly achieve lower costs of enzymatic DNA synthesis, as recently reported by Tang et al. This strategy allowed the enhancement of the coding rate to more than log_2_3 per unit time and avoidance of deletions.^45^ In the future, automation^39^ of the reading, writing, and operative procedures, as well as the future developments of microfluidics, may forward DNA data storage toward a reduction of its economic costs.^12^

#### 2.6.2 Issues Related to the Process Time Scales

Besides economic costs, automation could possibly lead to a reduction of the time costs for DNA data storage, as well. Indeed, the time requirements for the process are another limiting factor in the development of DNA data storage. For example, the reading speed is much lower than standard silicon-based storage media.^97^ This could be detrimental, especially when the only possible alternative to retrieve a file would be to read the entire database: it would be a very slow process. For these reasons, DNA data storage systems have been proposed for long-term archival purposes^97^ that need infrequent reading, while future investigations will be needed to fully realize random access.^78,75,70^

Conversely, in regards to nanopore reads of labeled DNA, each label is read in [10^–1^; 10^1^] ms.^101,21,102^

The writing speed of DNA data storage is lower than that of standard technologies, too. The current writing speed for DNA archives is in the order of kilobytes/second, thus a reading/writing cycle has a significant cost in terms of time.^8^ It is estimated that DNA data storage will need writing speeds in the order of gigabytes/second to be comparable with commercial cloud storage systems in around 10 years. This means DNA data storage must fulfill a gap of 6 orders of magnitude in regard to the writing (i.e., synthesis) and a gap of 2–3 orders of magnitude in regard to the reading (i.e., sequencing).^12^

In order to enhance the read/write speed of DNA data storage, one of the goals should be to make it suitable for frequent data reads and modifications. This is another pivotal reason for the investigations about synthetic polymers as data storage tools, together with the mentioned high cost of DNA.^97^

While writing and reading operations regarding DNA-stored data need to be improved, when it comes to preservation time, DNA is better than current storage technologies. Indeed, the maximum preservation time of information is 50 years for digital memories and 500 years for paper, while it is millennia for inorganic matrix-encapsulated DNA.

In conclusion, DNA data storage presents both advantages and disadvantages with respect to traditional storage methods regarding costs. It is also for this reason that research interest is growing in this field.

## 3 Structure-Based DNA Data Storage

### 3.1 DNA Nanotechnology Versus Synthetic DNA Sequence for Digital Data Storage

**Figure 8:**
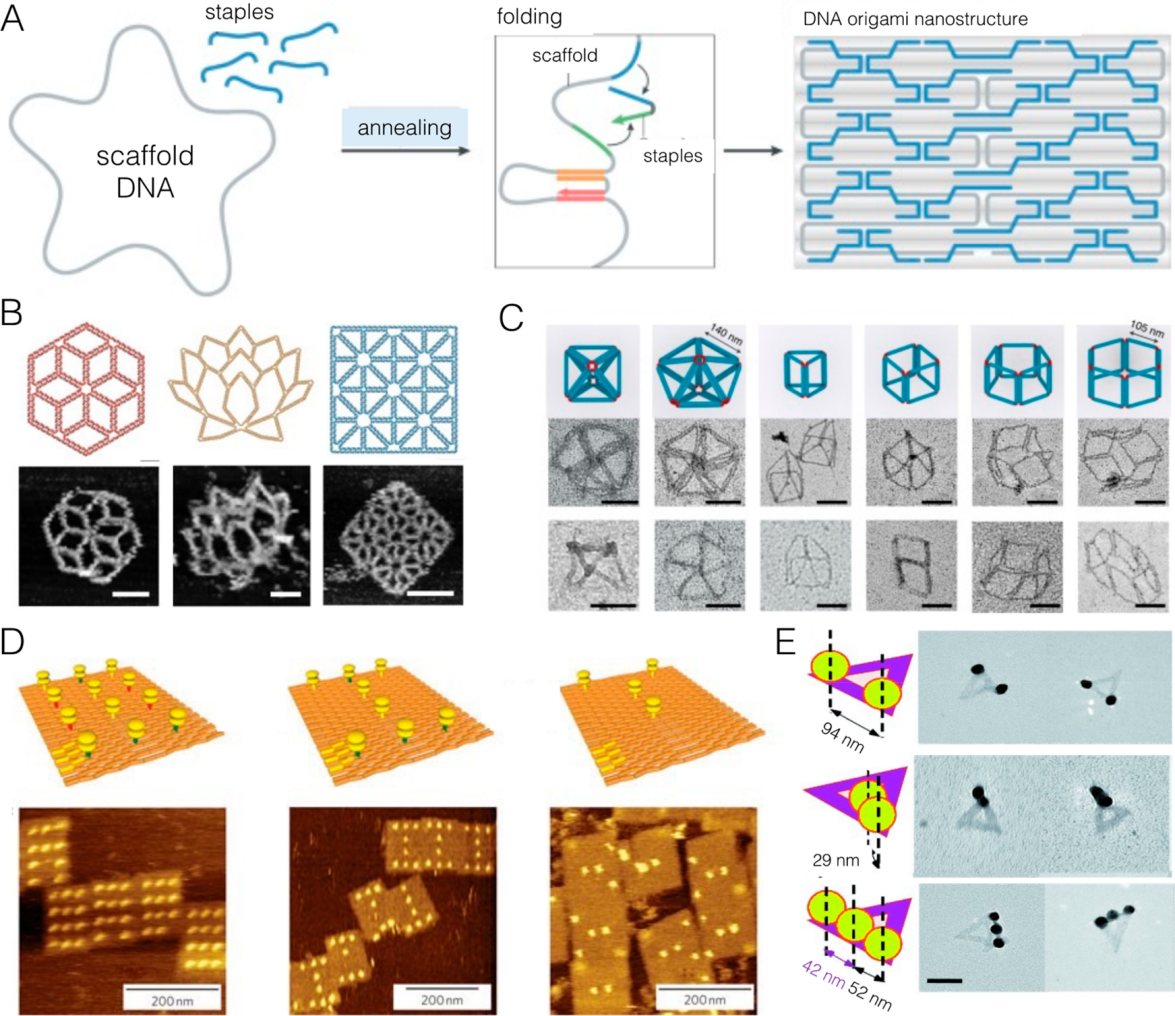
DNA nanostructures are data storage architectures. (A) DNA origami leverages the specific base-pairing motifs of DNA to create arbitrary structures. When a long scaffold strand (several thousand nucleotides in length) is combined with hundreds of short “staple” strands, complementary regions on the different strands will hybridize, thereby folding the scaffold into a desired conformation. These structures can then be examined using (B) atomic force microscopy or (C) electron microscopy, for example. (D) Data can be written onto DNA origami sheets through the site-specific addition of proteins; the data may be read using AFM. (E) Nanoparticles can also be controllably positioned on DNA origami with nanometer-scale resolution, which enables data writing with cryo-EM readout. (A) Image reproduced with permission from ref (108). Copyright Springer Nature 2021. (B) Image reproduced with permission under a Creative Commons Attribution 4.0 License (CC BY) from ref (109). Copyright 2019 AAAS. (C) Image reproduced with permission from ref (110). Copyright 2020 Springer Nature. (D) Image reproduced with permission from ref (111). Copyright 2010 Springer Nature. (E) Image reproduced with permission from ref (112). Copyright 2010 Wiley-VCH.

DNA nanotechnology may also be employed to overcome the limitations illustrated above in synthesis and reading. Because of the self-assembled nature of DNA nanostructures (Figure 8), it is possible to significantly reduce the synthetic demand and to eliminate the need for next-generation sequencing for DNA data storage. DNA nanotechnology leverages the unparalleled molecular recognition motifs of the nitrogenous bases to create arbitrary two- and three-dimensional structures from the self-assembly of user-defined DNA strands.^24,103^ Through careful design of the sequences of these strands, which can be easily synthesized in an automated manner or even purchased from commercial vendors, exquisite control over their final assembly can be realized, thereby enabling the construction of nanoscale shapes and patterns. The main approaches in structural DNA nanotechnology can be divided into three groups: DNA origami, DNA tile assembly, and wireframe DNA structures,^104^ all of which have been extensively reviewed elsewhere.^103^ Among these, DNA origami is the most widely used method for the construction of DNA-based data storage structures at the nanoscale. Importantly, all of these bottom-up approaches enable the production of asymmetric patterns, which is a key criterion for data storage applications: instead of encoding information directly into the sequence of bases, data may be stored in the three-dimensional shape of these assemblies.

Because of the noncovalent nature of DNA nanostructures, they can be reconfigured using established strategies, including strand displacement,^26^ thermal annealing,^105^ and pH changes.^106^ The reversible Watson–Crick base pairing means that, unlike data encoded directly into the primary DNA sequence, data storage platforms based on DNA nanostructures can be “erased” and “rewritten” multiple times without requiring any laborious chemical synthesis, which decreases the synthetic demand and cost associated with these methods.^25^ Additionally, the reconfigurable nature of these constructs enables their use in data operations and computation, analogous to existing computer memory systems. Because each bit is formed through self-assembly, it is also possible to encrypt information by initially omitting a key element from the assembly mixture; only upon addition of the correct “password” molecule can the DNA-based data be “read.”

Compared with encoding data within the nucleotide sequence itself, data storage based on DNA nanotechnology has one major drawback: data storage density. While data written directly into the DNA sequence theoretically allows 1 exabyte (or 1 billion gigabytes) to be stored in every cubic millimeter of DNA,^107^ the data density that has been attained so far using DNA secondary structure is much lower because it requires ∼100 base pairs per bit.^25^ That being said, this density is still approximately 3 orders of magnitude higher than current hard drive technologies, with further improvements conceivable through the optimization of the 3D DNA structure. Considering the advantages of encoding information into the secondary structure—including ease of readout, synthetic simplicity, and reconfigurability—this is a minor obstacle and one that may be mitigated through the careful design of DNA nanostructures.

### 3.2 DNA Nanostructure-Based Information Storage Platforms: Assembly and Readout

When comparing DNA nanostructure data storage to traditional sequence-based methods, the major differences lie in the reading and writing steps. In particular, standard DNA data storage requires slow and costly DNA synthesis, while DNA nanostructures already store molecular data in two- and three-dimensional objects. In fact, the assembly of DNA origami is, itself, a molecular information encoding process, wherein the long scaffold strand is folded with hundreds of short “staples” to form a predetermined structure (Figure 8). The size and morphology of the resulting structures can be assessed using various ensemble and single-molecule characterization methods, thereby enabling the readout of information stored in the shape and structure of these nanoscale assemblies. The use of this suite of techniques (described in detail in the following sections) has two major advantages: (1) Depending on the design and the physical attributes of the data storage structure, it may be possible to perform more than one type of characterization. Comparing the results of different readout methodologies may allow for the identification of systematic biases in each modality, which generates a feedback cycle wherein structures may be improved upon and recharacterized. (2) The identification of larger structures (on the order of approximately tens of nanometers) de facto requires lower resolution than the differentiation of single bases, thereby facilitating the use of less precise techniques without sacrificing accuracy. Additionally, because the single-molecule readout methods used for the assessment of DNA nanostructures are also used in DNA sequencing, these techniques are constantly improving: in this way, the advancement of sequence-based DNA data storage also supports the growth of alternative, structure-based approaches.

#### 3.2.1 Gel Electrophoresis

**Figure 9:**
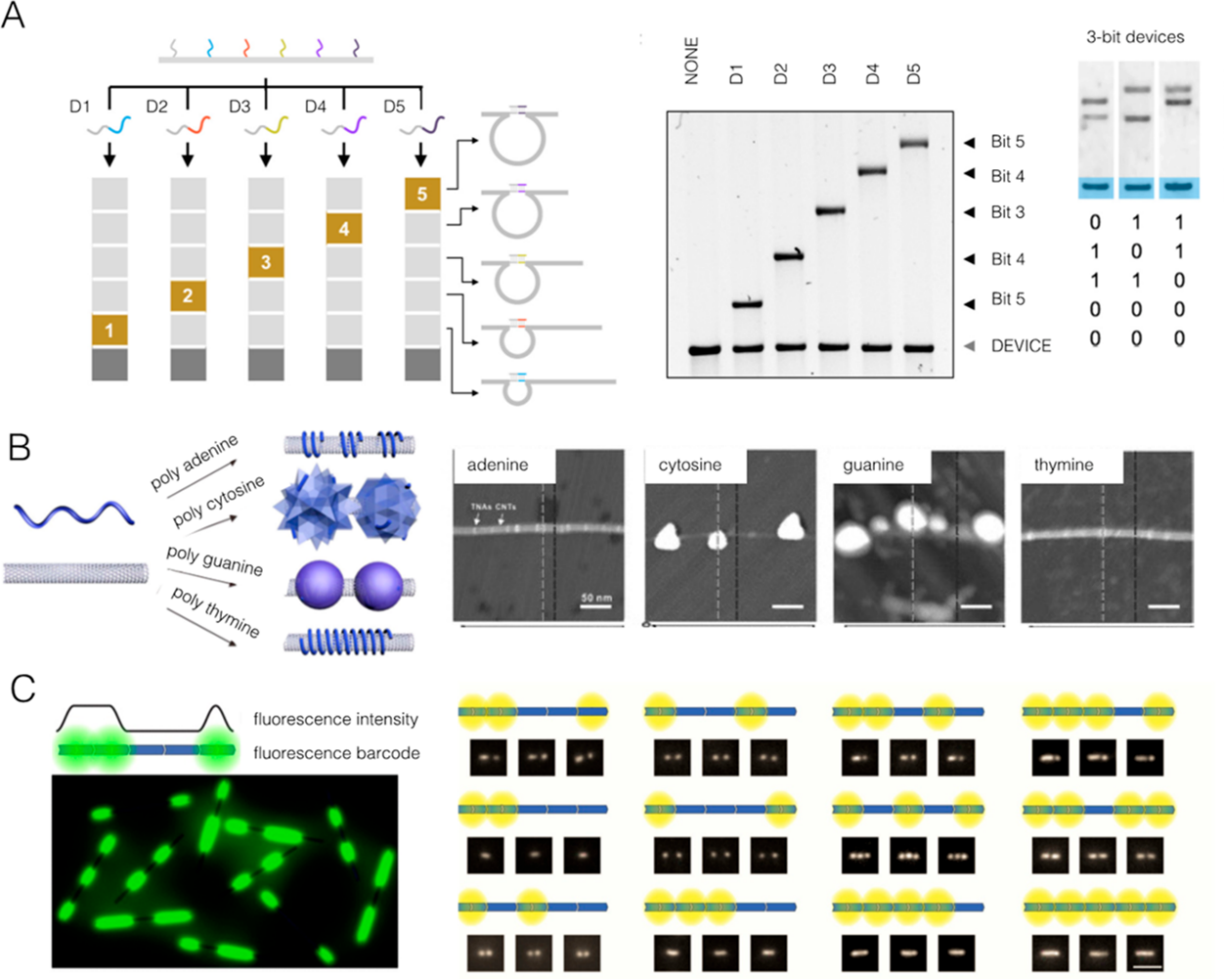
Examples of DNA nanostructures for digital information storage. (A) The folding of DNA origami into loop structures upon binding of a biomolecule target generates a shift in the assembly’s electrophoretic mobility. Image adapted with permission under a Creative Commons Attribution 4.0 license (CC BY) from ref (114). Copyright 2017 Oxford University Press. (B) The association of different DNA sequences to carbon nanotubes produces an array of morphologies and, therefore, can be used to produce barcodes. Image adapted from ref (116). Copyright 2019 American Chemical Society. (C). Data strings based on regions of varying fluorescence intensities along a DNA nanotube can be read out using single-molecule fluorescence microscopy. Image adapted from ref (117). Copyright 2021 American Chemical Society.

A first and very simple method to read data is the use of gel electrophoresis, which remains one of the key methods to differentiate DNA nanostructures of different shapes and sizes, as well as to assess their yield. Through the formation of DNA nanostructures with prescribed differences in size, it is possible to encode information and then read this out using the discrete bands formed on a gel. To this end, simple structures involving hairpins, loops, or G-quadruplexes placed along linear DNA backbones can also be used to store digital data. For example, Halvorsen and Wong used the change between a closed loop structure (“1”) to a linear structure (“0”)—which have different elution times by gel electrophoresis—as a binary switch. The authors used electrophoresis to demonstrate the readout of an 11 byte ASCII message.^113^ The creation of many loops of different sizes, each distinguishable by gel electrophoresis, offers a greater number of possible bits in each lane (Figure 9A).^114^ The formation of loop structures is not the only operation of DNA nanostructures that can be directly probed using gel electrophoresis. In an alternative approach, five single-stranded nucleotides were annealed together to form an assembly with three addressable overhangs; when complementary strands to each of these overhangs were introduced, the site changed from a “0” (single-stranded) to a “1” (double-stranded) state, which could then be reversed using strand displacement.^115^ These examples highlight the simple and inexpensive nature of gel electrophoresis as a readout platform, especially when compared with optical, electrochemical, and AFM-based methods. However, the relatively long read times and low data capacity of these methodologies limit their applicability. Gel electrophoresis, being a bulk measurement, also requires substantial quantities of DNA for readout relative to single-molecule methods like AFM, electron microscopies, and nanopore techniques.

#### 3.2.2 Fluorescence

Bulk fluorescence measurements can read out data encoded into DNA nanostructures. In an early example, DNA strands were used as “molecular memory” by transitioning thermally between a hairpin structure (unwritten state) and a duplex structure (written state).^118^ The oligonucleotides were appended with fluorophore/quencher pairs; as the thermal cycling occurs, the fluorescence output reversibly switches between two defined states to produce a binary signal. Unfortunately, because this process is performed in solution, the whole memory is erased simultaneously, which highlights the need for alternative strategies that enable spatial addressability. To this end, single-molecule fluorescence methods may be used instead to read out DNA origami breadboards appended with fluorophores. In one approach, termed “polychromic address multiplexing,” DNA origami was separated into spatially resolved “cells,” each of which contained a set of fluorophores appended to DNA. Some of these linkers contain photocleavable groups, which enables the disruption of energy transfer processes between adjacent dyes, thus resulting in a fluorescence change. The switch between two possible intensity values provides the binary logic in this system.^119^ Through the use of single-molecule total internal reflection fluorescence (TIRF) microscopy, it is possible to decode fluorescent barcodes assembled on DNA nanostructures.^120^ Pan et al. utilized this diffraction-limited imaging technique to devise a method to group fluorophores into bright (“on”) lengths along a DNA origami rod.^121^ Such bright spots were separated by dark (“off”) regions to create geometric barcodes using only one color of emitter (Figure 9C). Another tactic used a DNA origami “breadboard,” which was divided into a grid of pixels or an “indexed matrix of digital information.” Each specific location on the origami represents a bit, with the presence (“1”) or absence (“0”) of a docking site for a fluorophore encoding binary information.^22^ Docking sites are located using DNA points accumulation for imaging in nanoscale topography (DNA-PAINT), a form of super resolution fluorescence imaging that relies on transient binding of short DNA strands to prepositioned sites on an origami structure.^122^ In this example, unique data patterns are created by selecting which staple strands within the origami possess data domains. This approach also uses error-correction algorithms that enable message recovery even when individual docking sites are missing. Unlike DNA sequencing, which requires multiple reads to reach a consensus, this tactic can read 750 origami to reach a 100% probability of full data retrieval, which means that only femtomoles of material are needed.

#### 3.2.3 Atomic Force Microscopy

Early examples of DNA origami were reported in the mid 2000s and involved the assembly of 2D arrays to form various images, including the letters of the alphabet,^123^ a nanoscale Mona Lisa,^124^ and a map of the Americas.^125^ Atomic force microscopy (AFM) was used to “read-out” images formed by DNA origami, and this remains a key technique for the study of DNA-based nanomaterials.^126^ AFM measurements detect differences in height over a sample surface, without affecting the sample, thus rendering this method ideally suited to reading out three-dimensional patterns on DNA origami. Binary information can be written by precisely placing nanoparticles or proteins at defined positions on a DNA breadboard. In the context of DNA data storage, Zhang et al. demonstrated in 2019^127^ a “DNA braille” system, which was prepared by patterning biotinylated overhangs onto DNA origami. The data in this system are encrypted; only when streptavidin is added and binds to biotin does the pattern become readable by AFM. The decryption time for this method is 1–2 h, including sample processing, imaging, and readout—this time could be reduced by using high-speed AFM methods and fully automated image analysis algorithms. Similarly, Fan et al. used AFM to decode information stored in DNA domino arrays.^127^ The use of DNA overhangs bearing streptavidin enables the use of strand displacement reactions to controllably erase and rewrite data on the DNA origami surface,^128^ thereby underlining the advantages of DNA nanotechnology as an information storage platform. AFM is also suitable to look at DNA positioned on other types of nanomaterials; for example, it was found that condensing DNA strands onto carbon nanotubes creates height differences that were observable by AFM (Figure 9B). Control of the patterning of these protrusions, which interestingly do not rely on DNA hybridization, may allow for the production of two-dimensional barcodes on carbon nanotubes.^116^

#### 3.2.4 Electron Microscopy

Relying on similar principles, the decoding of DNA nanostructures can also be achieved using electron microscopy (EM). DNA itself can be difficult to visualize using EM because of insufficient electron density-related contrast, and therefore, often requires staining. As such, EM is better suited to the examination of hybrid structures, wherein the DNA is used to create “barcodes” made of gold nanoparticles,^129^ for example. Different barcodes can then be used to track the cellular uptake of various nanostructures because EM allows for the identification of subcellular compartments. EM exhibits some of the same advantages and pitfalls of AFM: while these techniques allow for high-resolution two- and three-dimensional images to be formed of DNA nanostructures, they are time-consuming and expensive, as well as relatively low-throughput. As cryo-EM and liquid-cell EM techniques continue to improve, the direct imaging of biomolecules might offer an alternative in the future with better resolution on the single-molecule level even without the use of staining or nanoparticles.

#### 3.2.5 Nanopore Measurements

**Figure 10:**
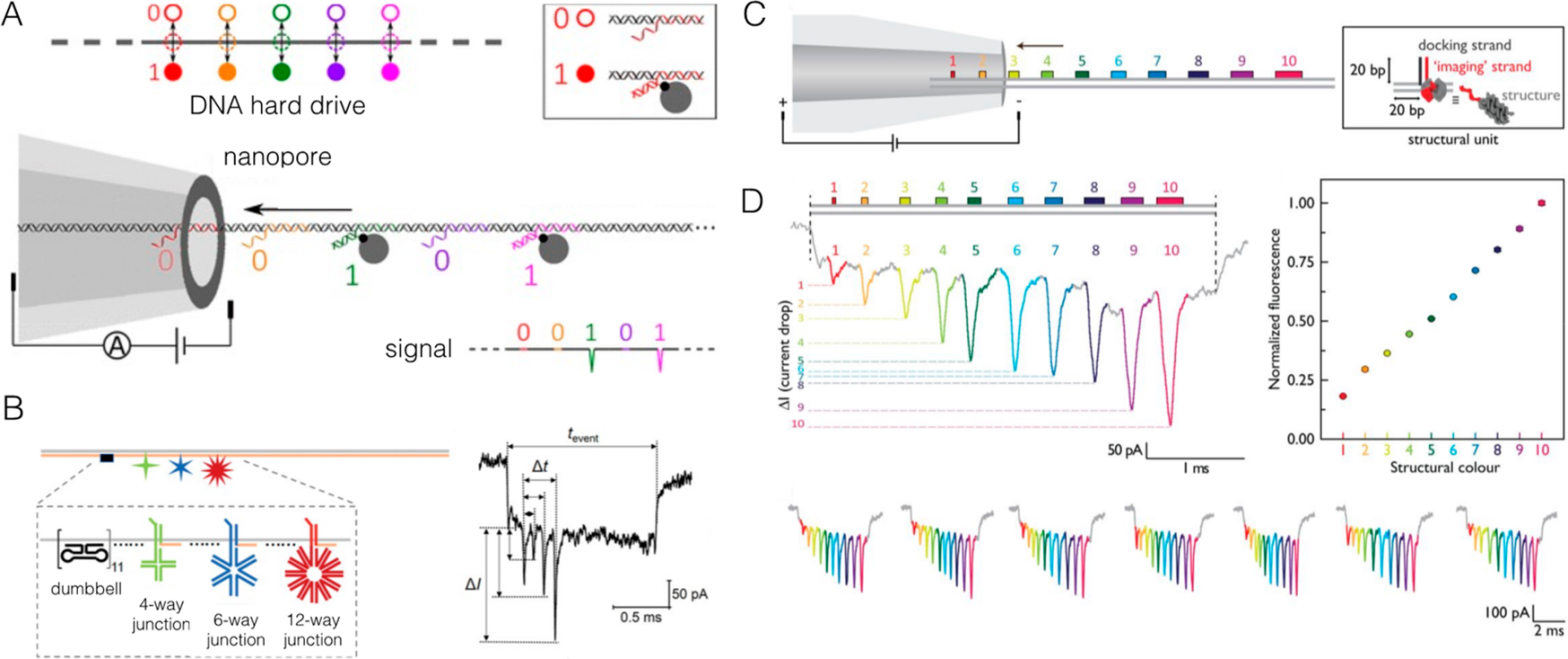
DNA data storage structures relying on nanopore readout. (A) An encrypted “DNA hard drive,” wherein readout may only occur once the correct molecular “keywords” have been added. Streptavidin molecules (gray circle in inset) partially block the nanopore as they translocate, which causes a momentary decrease in the current. Image reproduced from ref (25). Copyright 2020 American Chemical Society. (B) Multilevel barcoding is achievable by exploiting DNA junctions with different sizes, which create current drops of variable magnitude. Image reproduced with permission under a Creative Commons Attribution 4.0 License (CC BY) from ref (102). Copyright 2021 Wiley-VCH. (C) A DNA barcode with “structural colors” can also be formed by closely packing structural units, which therefore read as one protrusion. These units may be based on either monovalent streptavidin or a DNA cuboid. (D) Nanopore microscope can be used to detect up to 10 structural colors within the same DNA data string. The correct identification of the “color” was verified using fluorescence microscopy, wherein fluorescently labeled (5′-fluorescein) structural units were used. (C,D) Images reproduced with permission under a Creative Commons Attribution 4.0 License (CC BY) from ref (130). Copyright 2022 Springer Nature.

More recently, through the use of long DNA backbones as in DNA origami, the organization of DNA protrusions has been used to produce three-dimensional DNA barcodes^21^ or hard drives that may be read using solid-state nanopores. Nanopore methods require no labeling for readout, which makes them an attractive alternative to fluorescence. Briefly, an electric field is applied across a nanoscale hole (made from glass or Si_3_N_4_, for example), which causes molecules to translocate through this nanopore. As the analyte passes, it modulates the ionic current signal because of its 3D shape blocking the pore—in this way, the structure of the DNA nanoconstruct is translated directly into an electrical signal (Figure 10). The resulting current–time traces can then be analyzed using automated methods, which allows for rapid data decoding.

The use of nanopores to read out digital information encoded in DNA nanostructures was demonstrated by Bell and Keyser, who fabricated “DNA barcodes” to capture proteins.^21^ The authors used conical quartz nanopores with diameters of ∼14 nm for a 3-bit barcode that could be assigned with 94% accuracy. Now, these quartz nanopores can read out DNA hairpins along a carrier strand with a density of approximately 1 bit per 30 nm—ca. 3 times the data density of conventional hard drives.^25^ One of the major benefits of this method is their high speed: a single “DNA hard drive” can be read out on the millisecond time scale using a quartz nanopore because of the superior signal-to-noise ratio when compared with DNA sequencing. Solid-state nanopores combined with DNA nanotechnology have since been used to save and encrypt a grayscale image.^102^ Streptavidin-labeled scaffolds can also be used to create a secure data storage system that requires the correct molecular “keywords” to decode the data within the structure (Figure 10A). Multilevel storage architectures have been achieved using different DNA junction sizes to create a quaternary encoding system (Figure 10B).^102^ Increased storage density beyond binary barcodes can also be achieved by creating blocks of repeating structural units that appear as a single protrusion within the nanopore, which creates “structural colors” to generate up to 10 data levels.^130^ Compared with fluorescence, sequencing, or gel electrophoresis-based strategies, single-molecule nanopore measurements require less material and enable faster data reading; through a combination of this technology with deep learning methods,^131^ real-time nanopore data analysis is attainable.

Another important feature is random access, as demonstrated in 2021 by Bošković et al.^101^ In their work, random access of DNA barcodes was performed by exploiting a modified PCR method to increase the number of the target DNA nanostructures. Indeed, DNA structural barcodes were annealed as short oligonucleotides containing protrusions on single-stranded DNA (ssDNA) scaffolds to form digital bits at precise locations. In these structures, DNA nicks were ligated to favor the copy of the barcode by PCR. Each of these structures had a noncomplementary end, which acted as a barcode-specific primer template for the random access of data.

#### 3.2.6 Alternative Approaches and Polymer Chemistries

The use of double-stranded DNA as a storage medium was also exploited in recent work by Tabatabaei et al.^72^ on DNA punch cards. This macromolecular storage technology was used to encode the information in the sequence of bases of the DNA strands by using their sugar–phosphate backbone, i.e., topologically. Indeed, a pattern of nicking positions was precisely realized on the backbone of native dsDNA, and here, information was encoded by means of absence (i.e., 0) or presence (i.e., 1) of nicks. On the basis of enzymatic modification of DNA, nicks enable adding several functionalities to the storage system, for example, single-bit random access, pooling, and in-memory computation. However, the DNA punch cards system was able to store only up to 14 kB of digital information. Therefore, additional research is foreseen toward scaling its costs.

DNA as a natural polymer is not the only solution for data storage technologies. Therefore, researchers started to look for alternative molecular storage platforms based on synthetic polymers. Synthetic polymers can be used to increase stability against chemical degradation while offering a wide range of base modifications. Although alternative DNA bases have been introduced, synthetic polymers could be prepared using a set of monomers with a wider set of codes which expands the alphabet for data encoding.^97^ First experiments reading single-stranded synthetic biopolymers indicate that the reading step can be performed with biological nanopores without the use of an enzyme slowing down the translocation.^132^ As an example, Cao et al. used informational biopolymers composed of a backbone of poly(phosphodiesters) with dideoxyadenosine at both ends, and engineered-aerolysin nanopores. The results suggest a path to single-bit resolution at least in short polymers, however machine learning and training are needed for the successful readout. The study suggests an alternative way to store information with high density. The idea to use the backbone of an organic polymer to store digital information is similar to the approach discussed for DNA nanostructures.

Apart from DNA, other organic molecules have been recently proposed.^133,134^ Two interesting examples are the use of peptide sequences for data storage, as reported by Ng et al.^133^ and the use of urethanes as reported by Dahlhauser et al.^134^ Unfortunately in both these cases, reading required the use of mass spectroscopy, with the consequent limitation in terms of costs and speed. Recent advances in nanopore-based readout of short peptide sequences^135^ may speed up developments in this area.^53^

### 3.3 DNA Nanotechnology for Molecular Computation

The storage of data in DNA is undoubtedly an exciting possible solution to our ever-expanding data storage needs. This technology may lead to future hybrid electronic–biomolecular computing systems in which some portion of the burden of data storage is supported by DNA encoding, which raises the question: “Can more of the computer system’s functions be carried out using DNA?” By reducing the time overhead of conversion to a digital format and directly undertaking data processing tasks with DNA-based computation, it may be possible to create molecular computing systems that are more efficient than conventional electronic analogues. Because of the noncovalent nature of DNA nanostructures, these materials are primed for use in molecular computation. A working prototype for a DNA computer was developed by Adleman in 1994,^136^ wherein he used a separation-based approach to calculate a Hamiltonian path in a graph with seven summits. This problem was particularly suited to a molecular computing approach because it is an NP-complete problem; while verification of a putative solution has a complexity that is linear with respect to the number of nodes, the path space search is exponential in complexity with respect to the same. In a DNA computer, however, each DNA molecule plays the part of a separate processor, which enables many parallel operations to be carried out in a small reaction volume. This strategy greatly accelerates the initial path search, as statistics predict that DNA constructs corresponding to every possible path should be produced upon mixing. The task is then reduced to one of selection and filtering by removing invalid paths. The Adleman experiment acted as a proof of concept: in practice, the process was more time- and labor-intensive than a conventional digital approach. Nonetheless, the possibility of a DNA-based computer inspired researchers to further develop Adleman’s method and to devise advanced and powerful general DNA computing solutions. Early experimental and theoretical work examining the possibilities of DNA computation was focused on this parallelization and the benefits that it offered with regard to efficiently solving other NP-complete problems.^137,138^ Recent work on DNA computation has moved away from such problems toward recreating deterministic logical operations, for example, addition^139^ and multiplication,^140^ with definite outcomes. Su et al. produced DNA logic cascades, which allows the buildup of a full adder, a 4:1 multiplexer, and then, they combined these with other logic circuits to produce a DNA arithmetic logic unit (ALU): the foundation of general-purpose processors.^139^ These applications demonstrate the methods that can be used to mitigate error in DNA computation, which arise from the leeway and tolerance of mismatch inherent in sequence-specific DNA hybridization.

**Figure 11:**
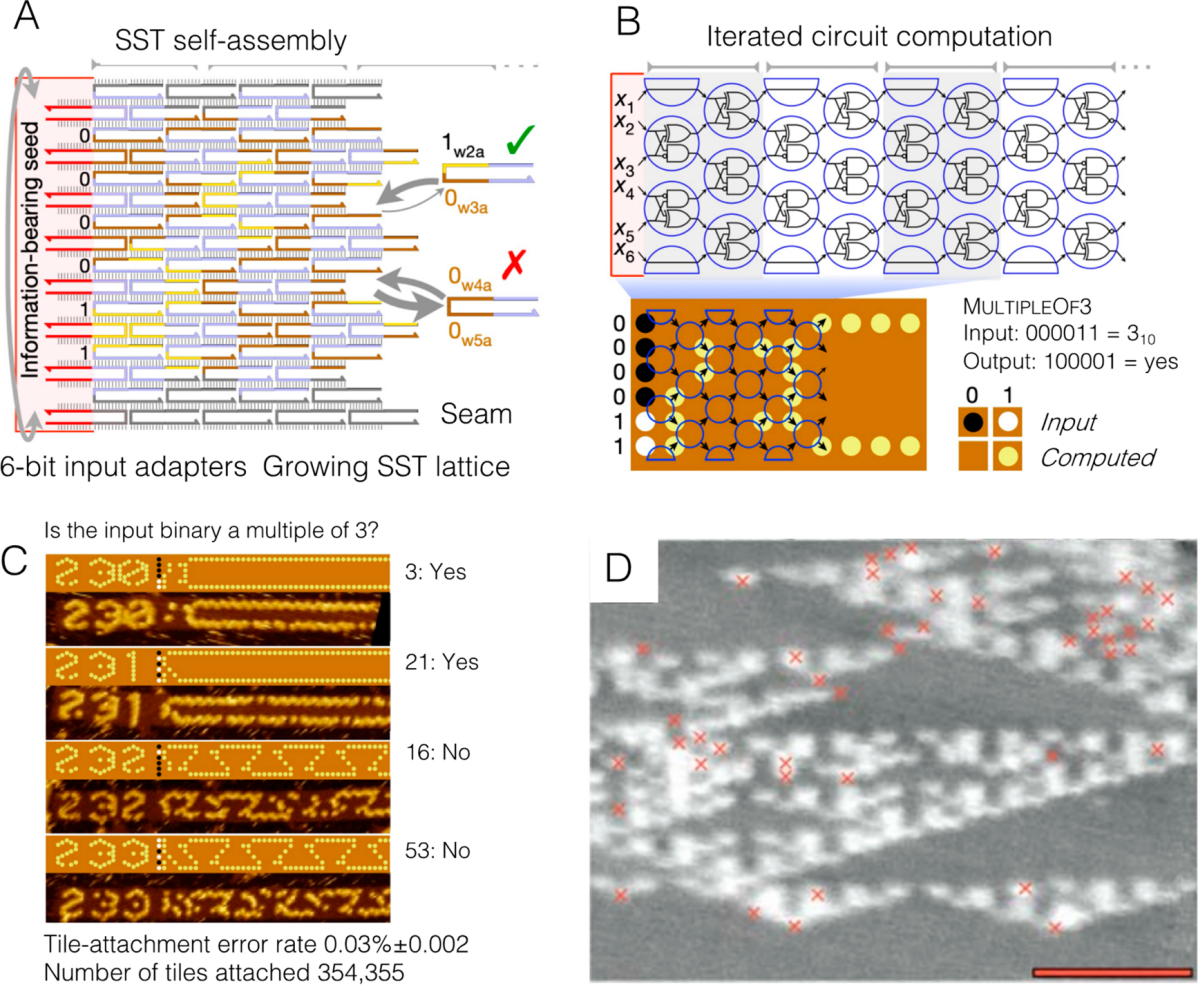
Tile-based computations and algorithmic self-assembly. (A) Self-assembly by SSTs. From a seed, tiles attach to the frontier of a growing SST lattice according to interaction rules determined by their exposed recognition sequences. (B) An iterated Boolean circuit mimicking the function of a computation to determine whether or not a binary number is a multiple of 310. A long enough lattice will settle into one or another fixed pattern corresponding to the calculation result. (C) The result of four “multiple of 3” tilings. The numbers at the left mark the experiment number. The tilings correctly determine which input numbers have a factor of 3. (A–C) Images adapted with permission from ref (144). Copyright 2019 Springer Nature. (D) A Sierpinski triangle created by a cumulative XOR computation performed by DNA tiles. Sierpinski’s triangle is a fractal pattern, and the self-assembly rule that creates it is Turing complete. Images reproduced with permission under a Creative Commons Attribution 4.0 License (CC BY) from ref (145). Copyright 2004 PLoS Biology.

Larger engineered DNA nanostructures show great promise for use in biomolecular computation as well as small origami structures. Robust, rigid DNA tiles with programmable “sticky ends” have been made using double-crossover (DX),^141^ triple-crossover (TX),^142^ and single-stranded tile (SST)^143^ motifs and used for a variety of algorithmic self-assembly experiments. This is facilitated by the logical equivalence of these tiles with Wang tiles, which are theoretical constructs with specified interactions that can simulate a Turing machine. A correctly designed, self-assembling set of these tiles is theoretically able to perform any computation that can be carried out by a conventional computer. Past applications of this idea include the design of a set of TX tiles that carry out a cumulative XOR operation, a set of DX tiles that self-assemble into a Sierpinski triangle, and impressively, a set of 355 SSTs that can be used to produce a variety of cellular automata capable of carrying out a number of computational tasks (Figure 11).^144^ Particularly interesting in the latter example is the ability to controllably reintroduce indeterminism by including a plurality of tiles that could fill a given niche and leaving the ultimately realized pattern up to competition. This brings about a marriage of the benefits of deterministic logic and the power of indeterministic computing to solve combinatorial problems, thereby highlighting the utility of DNA nanotechnology not only for data storage but also for molecular computation.

## 4 Conclusions and Outlook

DNA data storage—both the sequence- and structure-based versions—offers the possibility of storing digital information at very high data density. This promise has led a large number of actors (public and private institutions, corporations, etc.) to invest on the quest for advanced methods and experiments. Although great advances have been made toward DNA data storage, it is not yet competitive against conventional storage technologies. Significant challenges need to be overcome, in particular regarding writing speed and, hence, cost. While stored data size has been markedly increased, the current record for DNA digital data storage is still around 200 MB, with single synthesis runs lasting about 24 h.^8,12^ Achieving the storage of TBs of data at a low cost is unattainable with the current techniques. Toward this goal, great efforts on the development of encoding schemes, writing and reading processes, and storage procedures are presently being made.^146,9,81,44,74,14^

As the chemical and enzymatic processes for making sequence-defined nucleic acids continue to improve, the cost and time associated with writing DNA-based information is continually decreasing. These improvements are particularly important for sequence-based storage, but they importantly reduce costs for structure-based approaches, as well. Additionally, as alternative chemistries emerge, including unnatural nucleotides^147^ and small molecules that can modulate the structure of DNA,^148^ the parameter space for structure- and sequence-based DNA data storage is continually expanding. Importantly, these chemistries not only widen the breadth of materials that can be produced, but also may further extend the lifetime of DNA sequences, as these modifications render DNA less recognizable to enzymes.

For data readout, DNA sequencing is rapidly advancing, but current methods would be incompatible with unnatural monomer units, which limits the scope of the methods. Furthermore, all current DNA sequencing techniques require molecular machines like polymerases, which set fundamental limits for the throughput per enzyme, thereby meaning there is an upper threshold on the rate of sequencing even with massive parallelization. Emerging, rapid approaches to establish polymer sequence or three-dimensional structure one molecule at the time will improve the competitiveness of DNA data storage. Both natural and chemically modified oligonucleotides, as well as hybrid nanostructures involving DNA and quantum dots or nanoparticles, may be read out using solid-state nanopores. The versatility of this methodology, which hinges on the possibility of finely tuning nanopore size, makes this an attractive avenue for the future characterization of both pure DNA and composite materials. Through the use of quantum dots or fluorescent dyes, nanopore readout may be also combined with optical techniques to reduce the readout error rate without requiring enzymes to slow translocation.^149^ While the use of higher order nanostructures or composite nanomaterials does sacrifice data density, the advantages of these methods are expected to outweigh this drawback. In terms of synthesis, DNA nanotechnology greatly simplifies assembly procedures and produces structures that can easily be reconfigured. Indeed, computation is a natural extension for DNA nanotechnology, especially considering the vast library of naturally evolved enzymes that nature uses to copy, change, and repair genetic information. The interface between these natural systems and DNA nanotechnology is an active area of research, which generates other possibilities for DNA data storage that leverage nature’s evolved machinery. We foresee that DNA nanostructures made for information storage will find audiences in cryptography, steganography, and other fields^4,58,116^ and that combining DNA data storage with data analysis techniques such as neural networks will afford opportunities in a growing number of sectors.^127^

Because of the long-term stability of DNA under appropriate storage conditions, we predict that archival storage will be the most valuable application for DNA data storage. In this cold storage setting, information would be infrequently accessed from a relatively static DNA database. Considering that long-term, archival storage^97^ operates over long time scales—decades, centuries, and possibly millennia—this application requires only infrequent access to the stored information, which substantially reduces the impact of reading costs and long read times associated with DNA data storage. While the long-term stability of DNA, itself, is firmly established, further studies on the lifetimes of noncovalently assembled DNA nanostructures will need to be conducted to ensure that data stored in these formats are not compromised over time. Specifically, encapsulation and retrieval of DNA nanostructures in silica beads and other matrices should be examined, as well as the readability of DNA nanomaterials after prolonged freezing. It is also important to mention that the preservation of DNA digital archives can be implemented using not only in vitro substrates but also in vivo approaches.^150−153^ As three-dimensional nucleic acid nanostructures have also successfully been produced inside cells,^154^ there is potentially important synergy between in vitro/in vivo DNA nanotechnology and data storage, which remains, as of yet, unexplored.

Even in the context of archival storage, a DNA database, like its electronic analogues, would benefit greatly from dynamic properties that allow data to be erased, rewritten, and updated. For example, in-storage file operations and computations, as well as the ability to repeatedly access DNA databases, would reduce DNA synthesis costs and abrogate the need to store multiple copies of archives. In this area, DNA nanostructures may present advantages over traditional sequence-based storage methods, as the reconfiguration of these supramolecular moieties is firmly established, though rarely in the context of information storage. The implementation of dynamic properties and a full characterization of the kinetics of these processes would bring DNA-based storage systems one step closer to practical viability.^78^

A combination of sequence- and structure-based approaches could represent a significant advancement to overcome the various hurdles associated with DNA data storage. For this field to reach its full potential, cooperation between scientists from a range of research areas will be essential to produce the advanced chemical techniques, instrumentation, characterization methods, and automated analysis tools that are required. As the wide range of topics, from mathematics to polymer chemistry, shows, data storage based on polymers will demand multidisciplinary consortia that ideally design the whole process from data encoding to decoding with a bottom-up approach.

## Vocabulary

**sequence-based DNA data storage**: storage approach in which information is stored in the nucleotide sequence of many individual DNA strands
**structure-based DNA data storage**: storage approach in which DNA is designed in a way that allows information to be stored in its structural features, e.g., 2D and 3D shape
**data encoding with error-correcting codes**: conversion from digital, binary data into the primary sequence (sequence-based) or 2D/3D structure (structure-based) of DNA by adding redundancy to counteract errors and partial data loss
**random access**: process by which a certain subset of information is selected (e.g., a single file) from a large pool of information
**reading**: process by which the data encoded in DNA are read; this process varies according to if the DNA data storage is sequence-based or storage-based
**decoding**: conversion of the information that was read from DNA into binary data; this conversion happens both in sequence-based and in structure-based DNA data storage
